# Dose-Dependent Effects of Myo-Inositol on Kainic Acid-Induced Epilepsy: Electrophysiological, Behavioral, Transcriptomic, and DNA Methylome Studies

**DOI:** 10.3390/ijms262211102

**Published:** 2025-11-17

**Authors:** Luka Kharkhelauri, Georgi Gamkrelidze, Veriko Bokuchava, Lia Tsverava, Eka Lepsveridze, Vincenzo Lagani, Merab Kokaia, Revaz Solomonia

**Affiliations:** 1 Institute of Chemical Biology, Ilia State University, 0162 Tbilisi, Georgia; luka.kharkhelauri.1@iliauni.edu.ge (L.K.); giga_gamkrelidze@iliauni.edu.ge (G.G.); veriko.bokuchava.1@iliauni.edu.ge (V.B.); lia.tsverava.2@iliauni.edu.ge (L.T.); eka_lepsveridze@iliauni.edu.ge (E.L.); vincenzo.lagani@kaust.edu.sa (V.L.); 2 Iv. Beritashvili Centre of Experimental Biomedicine, 0160 Tbilisi, Georgia; 3 Biological and Environmental Sciences and Engineering Division, King Abdullah University of Science and Technology, Thuwal 23955, Saudi Arabia; 4 Epilepsy Centre, Department of Clinical Sciences, Lund University Hospital, SE-221 00 Lund, Sweden

**Keywords:** epilepsy, epileptogenesis prevention, kainic acid, myo-inositol, transcriptome, DNA methylome, glutamate receptors, sodium channel

## Abstract

Epilepsy is a prevalent neurological disorder characterized by spontaneous recurrent seizures (SRS). Epileptogenesis is a multifaceted pathophysiological process that transforms a normal brain into one prone to chronic seizures. Targeting epileptogenesis is a compelling line of epilepsy therapy. Thus, discovering new drugs that oppose, mitigate, or modify epileptogenesis is a significant challenge in modern neuroscience. Our previous work demonstrated that, in a kainic acid (KA)-induced post-status epilepticus model, 28 days myo-inositol (MI) treatment reduces frequency and duration of motor and electrographic SRS even following cessation of treatment, for the following 4 weeks and identified MI as a promising antiepileptogenic compound To further evaluate the dose-dependent efficacy of MI, we applied the same experimental model using 30 mg/kg (dose used in earlier studies), 60 mg/kg, and 120 mg/kg to assess effects on hippocampal electrographic and motor SRS, as well as KA-induced spatial learning and memory impairment in a Morris water maze test. We found that MI had long-lasting, dose-dependent suppressive effects on behavioral and electrographic manifestations of epileptogenesis and ameliorated spatial learning and memory deficit induced by SE, with 60 mg/kg emerging as the most effective dose. Furthermore, we investigated transcriptomic and epigenetic alterations associated with the optimal MI dose and identified multiple affected pathways in the hippocampus. Interestingly, MI treatment resulted in transcriptomic upregulation and prevention of downregulation of several ion channel subunits, including GRIK3 and GRIN3A (kainate and NMDA receptor subunits) and the sodium channel subunit SCNB4. The obtained data highlight new molecular targets for epilepsy therapy and support the translational potential of MI.

## 1. Introduction

Epilepsy is a heterogeneous neurological disease characterized by spontaneous recurrent seizures (SRS). The immediate cause of epilepsy is a pathological increase in the excitability of neuronal networks that regularly induces abnormal hyper synchronization of neuronal activity, resulting in seizures [1]. 

Currently, about 1% of the world population suffers from this illness. Despite considerable efforts, the treatment of the disease largely remains symptomatic. In fact, existing antiepileptic drugs (ASMs) suppress seizures rather than modify the underlying mechanisms of the disease or provide a cure [2]. Thus, discovering new pharmacological agents that target the fundamental mechanisms of epilepsy is a challenge in modern neuroscience.

Epileptogenesis, triggered by various disruptive events, is a multifaceted, gradual process involving molecular, cellular, and network-level changes that transform a normal brain into an epileptic one [3]. The maladaptive regulation of neuronal excitability homeostasis has been identified as a key factor in epileptogenesis [4]. Epileptogenesis is relatively well studied in the context of acquired temporal lobe epilepsy (TLE), which is the most common form of epilepsy. TLE is a partial syndrome in which seizure activity originates in the temporal lobe of the forebrain, often in the hippocampus.

In animal models and human studies, it has been shown that numerous changes occur in the hippocampus during TLE epileptogenesis, in particular: loss of interneurons and pyramidal neurons, inflammation, ion channel and receptor expression modification, mossy fiber rewiring, transcriptome and epigenetic changes, synaptic transmission change, epileptiform and seizure activity development [3,5]. Currently, no widely accepted treatment that attenuates epileptogenesis is available. Nevertheless, targeting epileptogenesis remains one of the most promising therapeutic strategies [2]. Therefore, identification of new pharmacological agents that can inhibit, mitigate, or reverse epileptogenesis is a highly rewarding line of translational inquiry.

In our early studies, it was shown that an extract of Aquilegia vulgaris, a plant used in oriental folk medicine as an antiepileptic remedy, contains substances that interact with gamma-aminobutyric acid A (GABA-A) receptors in vitro [6]. These compounds were found to be myo-inositol (MI) and sleep-inducing lipid oleamide. Subsequent experiments using purified commercial MI and oleamide showed that MI completely inhibited radioactive muscimol (a GABA-A receptor agonist) binding to rat brain cell membranes, while oleamide approximately doubled 3H-Flunitrezepam (a specific ligand for the GABA-A receptor benzodiazepine site) binding, suggesting that MI may be a GABA-A receptor agonist, while oleamide functions as a positive allosteric modulator of GABA-A receptors [6]. GABA-ergic neurotransmission is the main inhibitory system in the forebrain, and its dysfunction plays a significant role in the development of epilepsy in general and ictogenesis in particular [1]. Consistent with this idea, our experiments showed that MI pretreatment significantly decreased the severity of acute seizures induced either by pentylentetrazolium (PTZ) or by kainic acid (KA) in experimental animals [7].

In our recent studies, using the rat kainic acid model of TLE, we identified MI as a promising antiepileptogenic drug. It has been demonstrated that MI alleviates molecular, cellular, systemic, and behavioral changes associated with epileptogenesis. Specifically, four weeks of MI treatment almost completely reversed KA-induced biochemical changes associated with epileptogenesis [8,9]. Furthermore, we demonstrated that in KA-treated rats, MI reduces the frequency and duration of motor SRS during and even 4 weeks after the treatment is ceased [10].

In addition, MI counteracts epileptogenesis-induced changes in miRNA profiles, mRNA levels, and expression of the sodium-MI transporter and LRRC8A subunit of the volume-regulated anion channel in the hippocampus [10,11]. Notably, in the same epilepsy model, it has been shown that MI decreases the frequency and duration of electrographic (EEG) SRS in the hippocampus, alleviates spatial learning and memory deficit associated with epileptogenesis, and attenuates cell loss in the hippocampus even 4 weeks after MI treatment termination [10]. In vivo, locally administered MI in the hippocampus suppressed evoked after-discharges in a dose- and time-dependent manner [12].

In the CNS, which has high MI content, MI is synthesized de novo and recycled in neurons. MI functions as an essential osmolyte and a precursor of phosphoinositides, which contribute to widely used signal transduction pathways in the CNS, regulating neuronal excitability [13]. Therefore, based on our data and the utility of MI-derived signaling pathways, we hypothesized that MI is an endogenous antiepileptogenic compound that contributes to neuronal excitability homeostasis [9,10].

In order to further refine the stated hypothesis, in the present study, we investigated the dose-dependent effects of MI on electrophysiological and behavioral markers of epileptogenesis in a KA-induced rat model of TLE. Thirty, sixty, and one hundred twenty mg/kg concentrations of MI were utilized in this study. The 30 mg/kg dose was previously identified as the threshold concentration for suppressing kainic acid–induced epileptogenesis in biochemical and behavioral experiments [9]. Based on reported MI brain concentrations [13], our calculations suggest that intraperitoneal injection of 30 mg/kg increases CNS MI levels by approximately 10–15%. Accordingly, 60 mg/kg and 120 mg/kg doses are expected to increase MI concentrations by 20–30% and up to 60%, respectively. The ‘high’ 120 mg/kg dose was chosen to remain tenfold below the known toxic concentration of intraperitoneally administered MI in rats [14], thus ensuring non-toxic but physiologically relevant effects

We found that MI suppressed motor and electrographic SRS in a dose-dependent manner and attenuated the associated decline in spatial learning and memory. In this regard, we identified 60 mg/kg as the optimal dose. Furthermore, we have shown that KA-induced epileptogenesis and MI treatment with 60 mg/kg were associated with extensive transcriptomic and DNA methylation alterations in the hippocampus, implicating several biological pathways. Importantly, MI treatment upregulated transcripts for several ion channel subunits (including GRIK3 and GRIN3A of the kainate and NMDA glutamate receptors, and the sodium channel beta subunit SCNB4), reversing their downregulation seen during epileptogenesis.

## 2. Results

### 2.1. Dose-Dependent Inhibition of Electrographic and Motor SRS and Interictal Spikes by MI

The frequency and duration of motor SRS, as well as electrographic SRS and interictal spikes (IIS) in the hippocampus, were measured in experimental animals injected with KA. Following KA-induced SE, the animals were treated for 28 days either with saline (KA+SAL group) or with one of three MI doses: 30 mg/kg, 60 mg/kg, or 120 mg/kg, forming the KA+MI 30 mg, KA+MI 60 mg, and KA+MI 120 mg groups, respectively.

**Figure 1:**
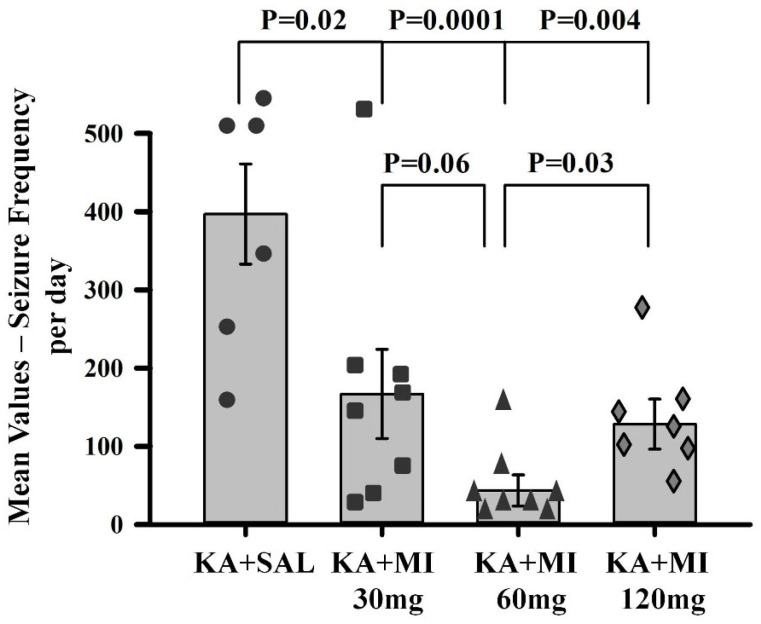
Mean number of electrographic SRS in the hippocampus of KA+SAL group and three MI-treated groups: KA+MI 30 mg, KA+MI 60 mg, KA+MI 120 mg. All MI groups exhibited significantly fewer electrographic SRS than KA+SAL, with the 60 mg/kg dose being most effective. Each bar represents mean values ± SEM. Individual values for each animal are shown on each bar.

**Electrographic SRS.** One-way ANOVA revealed a significant effect of treatment on electrographic SRS frequency in KA-injected animals (F3,27 = 9.93, *p* = 0.0001). Tukey’s test showed that the mean number of seizures per animal per day was significantly lower in all MI-treated groups compared to the KA+SAL group (*p* = 0.02, *p* = 0.001, and *p* = 0.004 for KA+MI 30 mg, KA+MI 60 mg, and KA+MI 120 mg groups, respectively) (Figure 1). Importantly, differences were also observed between MI-treated groups. The mean frequency of seizures was significantly lower in the 60 mg/kg group compared to the 120 mg/kg (*p* = 0.035) and 30 mg/kg (*p* = 0.06) groups. No differences were observed between the 30 mg/kg and 120 mg/kg groups. Thus, the antiepileptogenic effect of MI on electrographic SRS frequency appeared to be dose-dependent, with maximum efficacy at 60 mg/kg.

**Figure 2:**
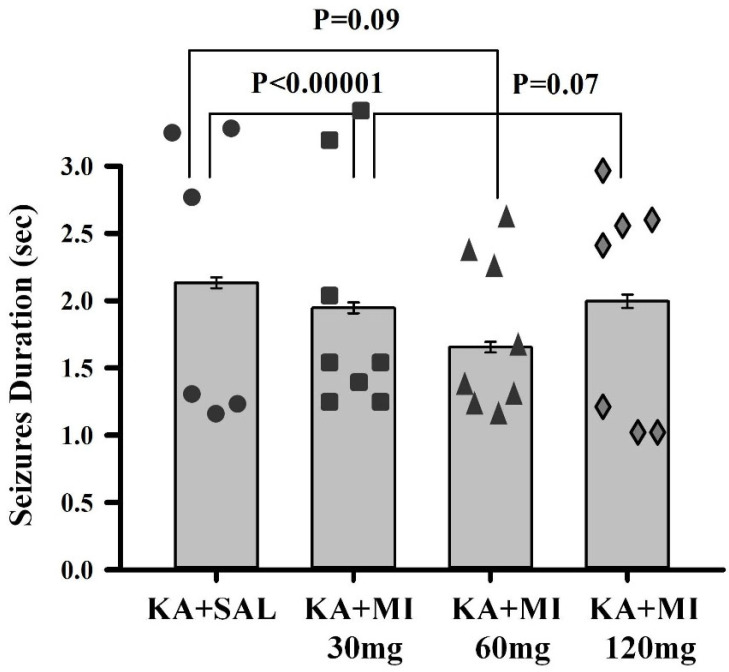
Mean durations of electrographic SRS in KA+SAL, KA+MI 30 mg, KA+MI 60 mg, and KA+MI 120 mg groups. Each bar represents mean values ± SEM. Individual values for each animal are shown on each bar.

**Electrographic SRS duration.** One-way ANOVA revealed a significant effect of treatment on electrographic SRS duration (F3,8383 = 9.79, *p* < 0.0001). Tukey’s test showed that the mean duration of electrographic seizures was significantly shorter in the KA+MI 30 mg group as compared to the KA+SAL group (*p* < 0.001) and minimally significantly less as compared to the KA+MI 120 mg groups (*p* = 0.07, Figure 2). The mean duration in the KA+MI 60 mg group was minimally significantly shorter compared to the KA+SAL group (*p* = 0.098) but not significantly different from the KA+MI 120 mg group (Figure 2). Thus, MI at 30 mg/kg had the strongest limiting effect on electrographic SRS duration, although a significant suppressive effect was also observed in the KA+MI 60 mg group. No effect was observed in the KA+MI 120 mg group compared with the KA+SAL group.

*Interictal spikes*

**Figure 3:**
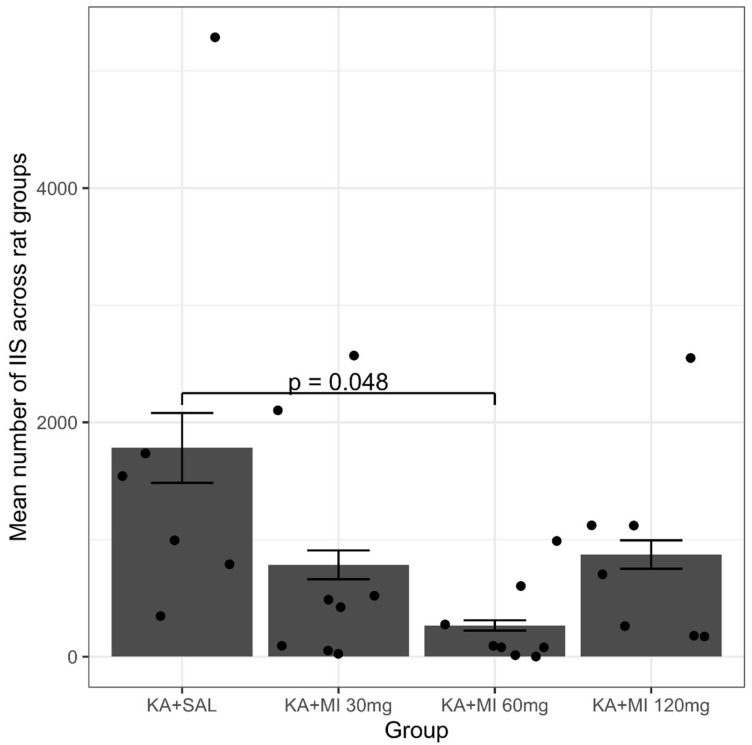
The mean number of IIS in different groups of rats. A significant decrease was detected only in the KA+MI 60 mg group. Each bar represents mean values ± SEM. Individual values for each animal are shown on each bar.

The interictal spikes (IIS) are closely associated with epileptogenesis. Analysis of the obtained results revealed that the data showed non-normal distribution (Shapiro–Wilk normality test W = 0.72634, *p* = 5.139 × 10^−6^). Further, Kruskal–Wallis multiple comparisons showed only one significant difference. Specifically, the mean number of IIS in the KA+MI 60 mg group was significantly smaller than in the KA+SAL group (adjusted *p* = 0.048, Figure 3). These results are in agreement with our previous data [10], where no significant difference was found in the mean IIS number between KA+SAL and KA+MI 30 mg groups.

### 2.2. Dose-Dependent Effects of MI on Motor SRS Mirrored MI Inhibition of Electrographic SRS

**Figure 4:**
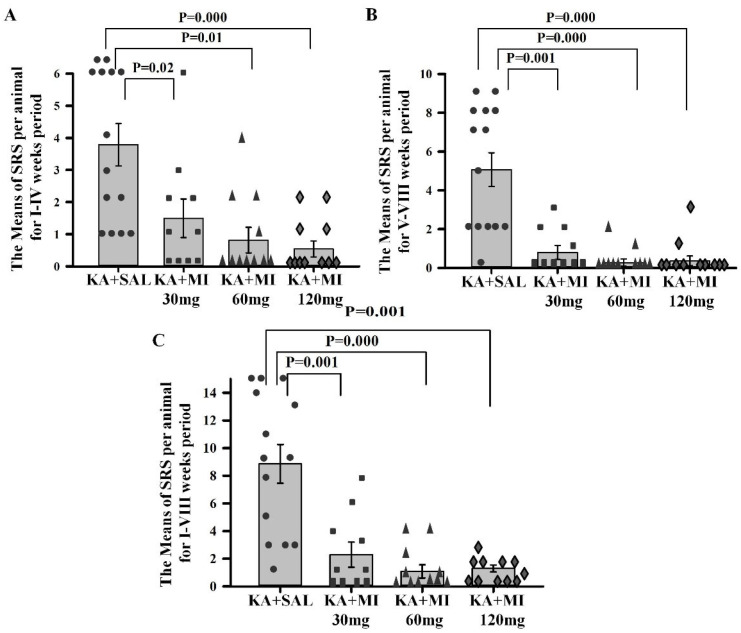
Mean number of motor SRS per animal during I–IV weeks (**A**); V–VIII weeks (**B**); and I–VIII weeks (**C**). All MI-treated groups exhibited significantly fewer motor SRS compared to the KA+SAL group. Each bar represents mean values ± SEM. Individual values for each animal are shown on each bar.

**Motor SRS frequency.** One-way ANOVA showed a significant effect of treatment on the mean number of motor SRS per animal during the first two separate 4-week time frames as well as during the combined 8 weeks of observation (F3,45 = 8.70, F3,45 = 8.70, F3,45 = 16.72; *p* = 0.0001 for all). All three doses of MI significantly decreased the mean seizure frequency compared to the KA+SAL group across all three analyzed periods (Figure 4, Supplementary Table S1). The mean number of SRS per rat during the 8-week observation period was nearly four times lower in each MI-treated group compared to the KA+SAL group (Figure 4). No significant differences were observed between MI-treated groups, although the mean SRS frequency values were numerically lower in the 60 mg/kg and 120 mg/kg groups compared to the 30 mg/kg group.

**Figure 5:**
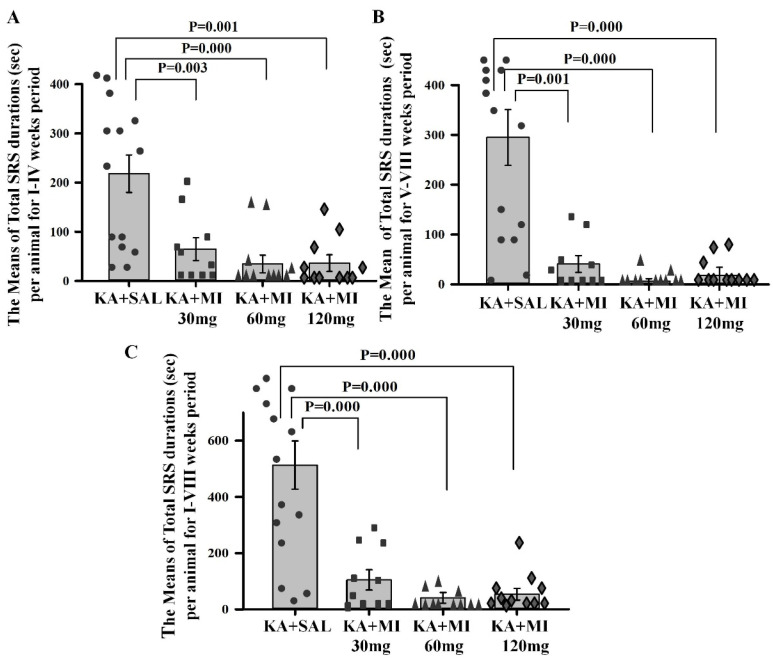
Mean duration of SRS (seconds) per animal during I–IV weeks (**A**); V–VIII weeks (**B**); and I–VIII weeks (**C**). All MI-treated groups exhibited significantly shorter motor SRS compared to the KA+SAL group. Each bar represents mean values ± SEM. Individual values for each animal are shown on each bar.

**Motor SRS duration.** One-way ANOVA showed a significant effect of treatment on SRS duration in the three recording periods (I–IV weeks: F3,45 = 10.93, *p* = 0.000; V–VIII weeks: F3,45 = 17.11, *p* = 0.0001; I–VIII weeks: F3,45 = 17.88, *p* = 0.000). Planned comparisons showed that the mean duration of SRS in all MI-treated groups was significantly shorter compared to the KA+SAL group across all three periods (Figure 5, Supplementary Table S1). There were no statistically significant differences between MI-treated groups; however, as with SRS frequencies, the rank order of means was: KA+MI 60 mg < KA+MI 120 mg < KA+MI 30 mg.

### 2.3. Dose-Dependent Effects of MI on Spatial Learning and Memory Task Performance After KA-Induced Epilepsy

**Spatial Learning.** Previously, we found that KA treatment of rats impairs their performance in the Morris Water Maze (MWM) spatial learning and memory tasks, while the administration of a minimal MI dose (30 mg/kg) mitigates the deficit [10]. Here, we investigated the dose dependence of the MI protective effect on spatial learning and memory. To this end, we tested KA- and MI-treated animal groups (KA+SAL, KA+MI 30 mg, KA+MI 60 mg, KA+MI 120 mg). In addition, control animals treated only with saline (CON+SAL) were also included.

The learning escape latency time two-way ANOVA analysis revealed that the effects of both factors, treatment and day, were highly significant (F4,128 = 15.86, *p* = 1.38× 10^−10^ and F3,123 = 9.73, *p* = 8.19 × 10^−6^, respectively). Tukey’s test showed that first-day mean escape time was significantly different from fourth-day mean escape times for CON+SAL, KA+SAL, KA+MI 60 mg, and KA+MI 120 mg groups (adjusted *p* values: 0.0017, 0.034, 0.049, and 0.017, respectively, Supplementary Table S2A). The difference for the KA+MI 30 mg group was minimally significant (*p* = 0.062).

**Figure 6:**
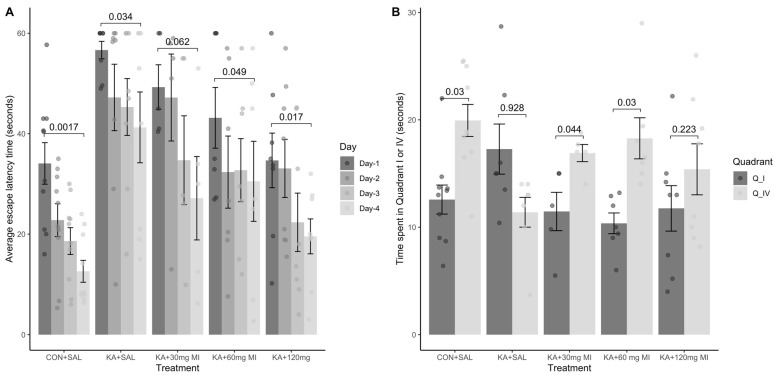
Spatial task learning and memory performance in MWM. (**A**) Distribution of the escape latency time (seconds, x-axis) separated by treatment group and day. Each bar represents mean values ± SEM. (**B**) Distribution of the time spent in quadrants I and IV (seconds, y-axis) separated per treatment group. Each bar represents mean values ± SEM. The control CON+SAL group, as well as two MI-treated groups (KA+MI 30 mg and KA+MI 60 mg), spent significantly more time in quadrant IV. Individual values for each animal are shown on each bar.

The control group CON+SAL showed good learning dynamics, as evidenced by a gradual decrease in mean escape latency time. Specifically, besides the significant difference between day 1 and 4, the mean escape latency on days 2 and 3 was significantly reduced relative to day 1 (*p* = 0.04, *p* = 0.01, respectively), and the latency period was significantly reduced on day 4 relative to day 2 (*p* = 0.008). However, there were no significant differences in latency between days 2 and 3, or between days 3 and 4 (Figure 6). Importantly, the KA+MI 120 mg group displayed similar learning dynamics: in addition to a significant difference in mean escape latency between day 1 and 4, there was a significant reduction in the latency period on day 4 relative to day 2 (*p* = 0.03) and a nearly significant reduction on day 3 relative to day 1 (*p* = 0.06). However, there was no significant difference in mean latency between the following pairs of days: 1 and 2, 2 and 3, or 3 and 4 (Figure 6A).

On the other hand, in groups KA+MI 30 mg, KA+MI 60 mg, and KA+SAL, in addition to the stated difference between days 1 and 4, there was no significant difference in the mean escape latency period in pairwise Tukey test comparisons of the training days (Figure 6). Finally, the comparison of the mean escape latency period on day 4 between the control (CON+SAL) and the other groups using a two-sample *t*-test revealed that the KA+MI 120 mg and KA+MI 30 mg groups were not significantly different from CON+SAL. By contrast, the KA+SAL and KA+MI 60 mg groups had significantly longer escape times than CON+SAL (*p* = 0.002 and *p* = 0.03, respectively). Importantly, the mean escape times of the KA+MI 30 mg and KA+MI 60 mg groups were not significantly different from the KA+SAL group, while the KA+MI 120 mg group exhibited a significantly shorter escape time compared with the KA+SAL group (*p* = 0.02). Thus, in the spatial learning task, the 120 mg/kg dose of MI displayed the highest efficacy among the tested doses in mitigating the adverse effects of KA-induced epileptogenesis on learning.

**Spatial memory task.** MI treatment also had a positive effect on the MWM memory task. The two-way ANOVA analysis revealed that both the quadrant effect and the interaction between treatment and quadrant were highly significant (F1,64 = 10.17, *p* = 0.002 and F3,64 = 4.85, *p* = 0.001, respectively). Tukey’s test showed that the control group CON+SAL, as well as the KA+MI 30 mg and KA+MI 60 mg groups, spent significantly more time in quadrant IV compared to the opposite quadrant (*p* = 0.03, *p* = 0.045, and *p* = 0.03, respectively, Figure 6B, Supplementary Table S2B). As in our previous study, no memory was formed in the KA+SAL group [10]. Despite high learning efficiency, no memory formation was observed in the KA+MI 120 mg/kg group.

To establish the specificity of KA and MI treatment on spatial learning and memory, we monitored the velocity of animal movement during training and memory tests. The velocity of the movement in the water maze was not significantly different among the groups, which indicates that the general health and motor system of the animals was in normal condition across experimental groups during water maze tests

In summary, the combined results from the analyses of electrographic SRS, interictal spikes, motor SRS, and spatial learning/memory performance indicated that the electrophysiological and behavioral manifestations of KA-induced epileptogenesis were most efficiently suppressed with an MI dose of approximately 60 mg/kg. Therefore, all subsequent molecular biology and biochemical experiments were performed with the MI dose of 60 mg/kg.

### 2.4. MI Had Profound Effects on Transcriptomic and Epigenetic Changes During Epileptogenesis in the Hippocampus

#### 2.4.1. Transcriptomics Analysis

**Figure 7:**
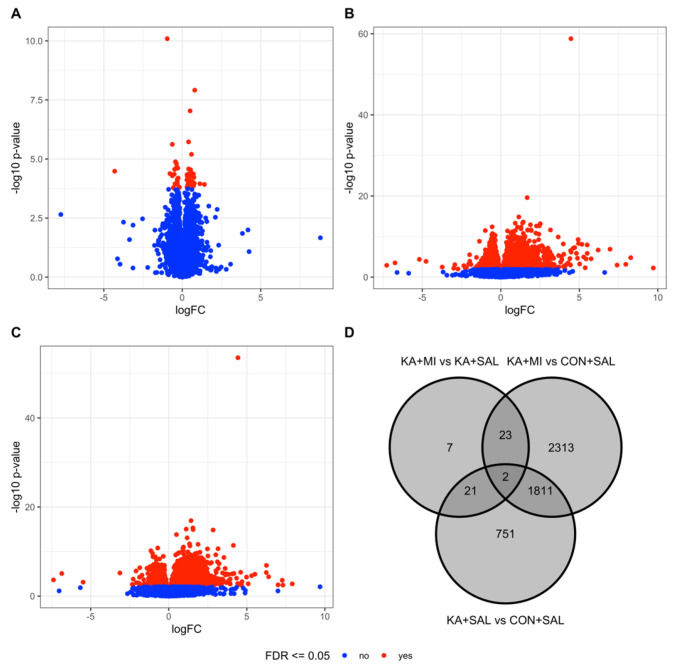
Differential gene expression analysis results. Panels (**A**–**C**) show volcano plots corresponding to the KA+MI vs. KA+SAL, KA+MI vs. CON+SAL, and KA+SAL vs. CON+SAL contrasts. The x-axis reports the log2-transformed fold change, while the y-axis shows the statistical significance as -log10 transformed *p*-value. Red dots correspond to significant transcripts (FDR-adjusted *p*-value ≤ 0.05), while blue dots represent non-significant transcripts. Panel (**D**) shows the Venn diagram summarizing the overlap between significant genes across the three contrasts.

Transcriptomic differences were detected between the two treatment groups (KA+MI, KA+SAL), as well as between each KA-treated group and control animals (CON+SAL). In particular, the KA+MI and KA+SAL groups differed significantly in the expression of 53 genes (Figure 7A,D, and Supplementary Table S3, FDR-adjusted *p*-value ≤ 0.05). 4149 unique genes were differentially expressed between the KA+MI and CON+SAL groups (Figure 7B,D, Supplementary Table S4) and 2585 between the KA+SAL and CON+SAL groups (Figure 7C,D, Supplementary Table S5).

**Figure 8:**
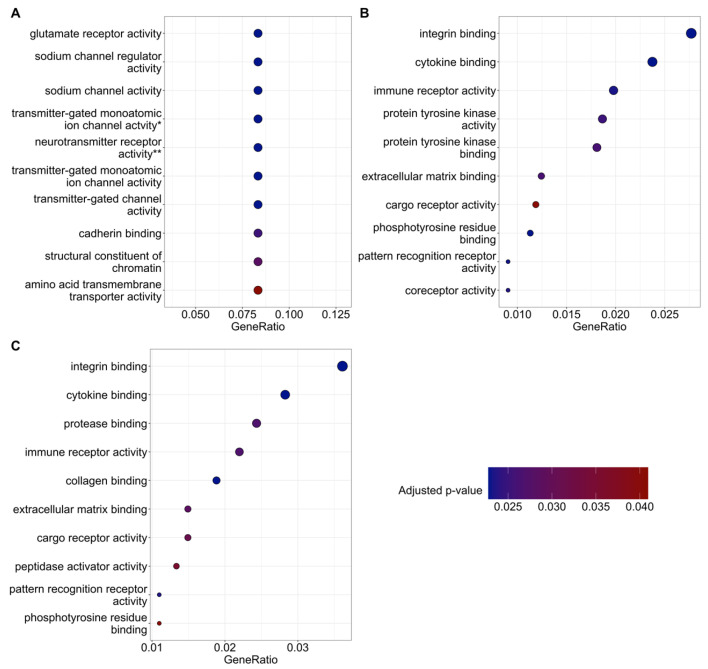
Gene Ontology (GO) biological processes with overrepresentation of upregulated genes. Panels (**A**–**C**) report the 10 most significant processes for the KA+MI vs. KA+SAL, KA+MI vs. CON+SAL, and KA+SAL vs. CON+SAL comparisons, respectively. Each dot represents a biological process; the x-axis reflects the proportion of upregulated genes, while the y-axis lists the process description. Dot size is proportional to the number of upregulated genes, while color represents the FDR-adjusted *p*-value. * transmitter-gated monoatomic ion channel activity involved in regulation of postsynaptic membrane potential. ** neurotransmitter receptor activity involved in regulation of postsynaptic membrane potential.

Interestingly, this relatively small list of 53 transcripts differentially expressed between the KA+MI and KA+SAL groups was enriched for 10 upregulated GO biological processes (FDR-adjusted *p*-value ≤ 0.05; Figure 8A, Supplementary Table S6), but none for downregulated processes (Supplementary Table S7). These upregulated biological processes included glutamate receptor and sodium channel regulator activity, as well as transmitter-gated ion channel activities implicated in postsynaptic membrane potential regulation. The other two contrasts (KA+MI vs. CON+SAL and KA+SAL vs. CON+SAL) presented larger numbers of both upregulated and downregulated biological processes (Figure 8B,C; Supplementary Tables S8–S11). The differences between these two contrasts mostly covered similar biological processes, but some were group-specific. For example, collagen and protease binding were specifically upregulated in the KA+SAL vs. CON+SAL group.

#### 2.4.2. Methylome Analysis

Numerous deregulated methylation sites (FDR-adjusted *p*-value ≤ 0.05) were identified for all three contrasts. All significant sites were located in the promoter or the first exon of known transcripts, hinting at a possible functional involvement.

**KA+MI vs. CON+SAL**

Contrasting KA+MI with CON+SAL resulted in 57 deregulated methylation sites (FDR-adjusted *p*-value ≤ 0.05, Supplementary Table S12). Hypermethylated sites were found in 7 genes (*Dnajc18*, *Hdac7*, *LOC102548917*, *Ubxn4*, *Rab6b*, *Prpf38b*, *Pigv*), while hypomethylated sites were found in 10 genes (*Them4*, *Ptges3l*, *Foxd2*, *Haspin*, *Rttn*, *Zfp717*, *Tiparp*, *Nes*, *Zfta*, *Slc33a1*).

Biological processes enriched for these genes included transcription and epigenetic regulation (Supplementary Tables S13 and S14). Interestingly, HDAC7 is involved in 14-3-3 protein binding activity, which in our previous study was identified as one of the inositol action targets [15].

**KA+MI vs. KA+SAL**

Contrasting KA+MI with KA+SAL resulted in 76 deregulated methylation sites (FDR-adjusted *p*-value ≤ 0.05, Supplementary Table S15). Hypermethylated sites were found in 8 genes (*Mtap*, *Tbc1d16*, *Smurf2*, *Gapvd1*, *Ublcp1*, *Prpf38b*, *Mcc*, *Pigv*), while hypomethylated sites were found in 10 genes (*Nes*, *Haspin*, *Foxd2*, *Arhgap24*, *Tiparp*, *Zfta*, *Rc3h1*, *Rttn*, *Zfp717*, *Slc33a1*). The list of biological processes for deregulated methylation sites is provided in Supplementary Tables S16 and S17.

**KA+SAL vs. CON+SAL**

Contrasting KA+SAL with CON+SAL resulted in 59 deregulated methylation sites (FDR-adjusted *p*-value ≤ 0.05, Supplementary Table S18). Hypermethylated sites were identified in 6 genes (*Arhgap24*, *Ubxn4*, *LOC102548917*, *Rab6b*, *Hdac7*, *Dnajc18*), while hypomethylated sites were identified in 8 genes (*Tbc1d16*, *Mcc*, *Gapvd1*, *Mtap*, *Smurf2*, *Ublcp1*, *Ptges3l*, *Them4*) (Supplementary Tables S19 and S20).

**Overlap between RNA-SEQ and Methylome studies**

There was overlap between RNA-SEQ and methylome changes, with several genes that were differentially expressed also showing differential methylation. These included:

- KA+MI vs. CON+SAL: *Zfp717*, *Ptges3l*, and *Hdac7*
- KA+SAL vs. CON+SAL: *Smurf2*, *Arhgap24*, and *Hdac7*

### 2.5. Quantitative PCR and Western Immunoblotting Experiments Confirm RNA-Seq Results

#### LncRNA-ENSRNOG00000064277 Was Increased in the Hippocampus of KA-Treated Groups

LncRNA-ENSRNOG00000064277 was drastically increased in the Hippocampus of KA+MI and KA+SAL groups as compared to the CON+SAL group (log2Fold Changes 2.45 and 2.84; adjusted *p* = 0.000000000434 and *p* = 0.000000000004, respectively, Supplementary Tables S4 and S5). We have further studied the expression of this RNA in the hippocampus and neocortex of KA+MI, KA+SAL, and CON+SAL groups by RT-PCR.

Hippocampus.

**Figure 9:**
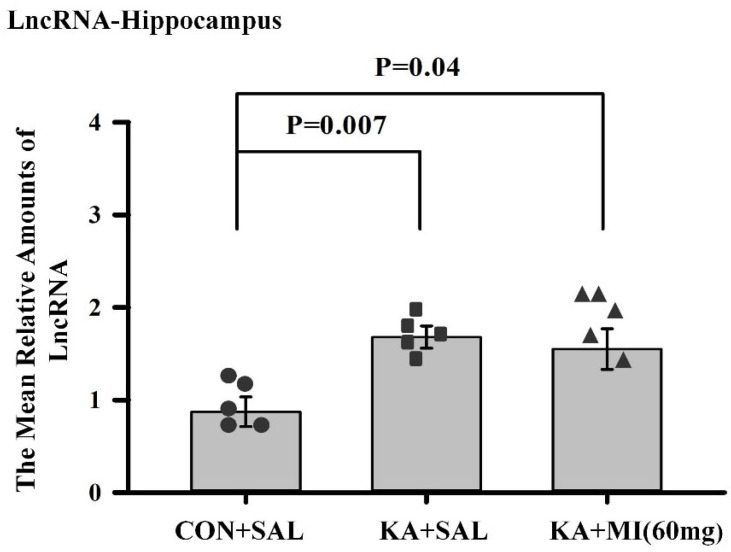
The mean relative amounts of LncRNA-ENSRNOG00000064277 in the hippocampus of three different groups of rats. The difference is significant between the KA-treated groups and the control group. Individual values for each animal are shown on each bar.

The effect of treatment was significant (One-way ANOVA F2,14 = 5.88, *p* = 0.017). The mean amounts of LncRNA-ENSRNOG00000064277 are higher in KA+SAL and KA+MI groups as compared to CON+SAL group (T = 3.64, *p* = 0.007, and T = 2.38, *p* = 0.044, respectively). For both comparisons, DF = 8. Figure 9.

**Neocortex**

No differences are significant (Supplementary Figure S1).

**Subcellular localization of LncRNA-ENSRNOG00000064277**

To determine its subcellular localization, the hippocampus was fractionated into cytoplasmic and nuclear components, followed by qRT-PCR analysis of the extracted RNA. LncRNA-ENSRNOG00000064277 levels were found to be at least 10 times higher in the cytoplasmic fraction (Supplementary Figure S2), suggesting its predominant cytoplasmic localization.

**Glutamate receptor subunits**

Comparison of biological pathways between KA+MI and KA+SAL groups has revealed that glutamate receptor activity was increased in the hippocampus of the KA+MI group (see Figure 8). Considering that MI treatment caused the decrease in SRS activities on electrophysiological and behavioral levels, and glutamate receptors are implicated in excitatory neurotransmission, apparently it looks controversial. We have further validated the detected differences.

GRIK3 (glutamate ionotropic receptor kainate type, subunit3) expression was affected by MI.

**Figure 10:**
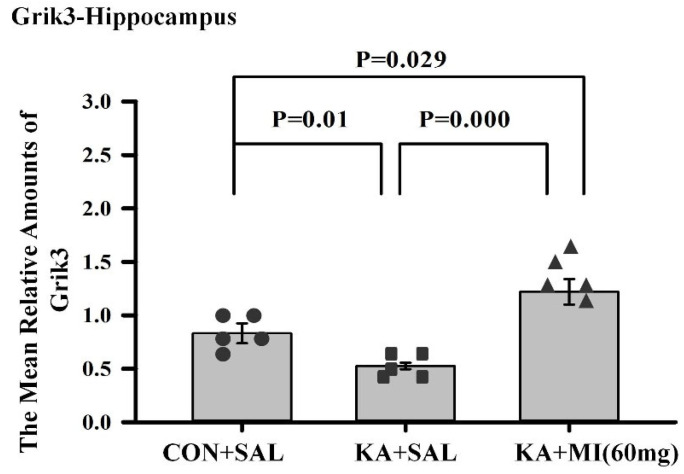
Mean relative amounts of GRIK3 mRNA in the hippocampus of CON+SAL, KA+SAL, and KA+MI 60 mg groups. Expression of GRIK3 was significantly less in the KA+SAL group as compared to both the CON+SAL and KA+MI groups. The level of GRIK3 mRNA in the KA+MI group exceeded that in the CON+SAL group. Individual values for each animal are shown on each bar.

One way ANOVA analyses indicated that the effect of treatment was significant (F2,14 = 15.61, *p* = 0.0001). The pair-wise comparisons with *t*-test showed that the amount of GRIK3 mRNA was significantly higher in KA+MI as compared to KA+SAL and CON+SAL groups (T = 5.69, *p* = 0.0001 and T = 2.65, *p* = 0.029, respectively. For both comparisons DF = 8). The GRIK3 mRNA was decreased in KA+SAL group as compared to CON+SAL group (=3.05, *p* = 0.016, DF = 8, Figure 10). Thus, MI treatment increased GRIK3 mRNA levels, whereas only KA induced epileptogenesis decreased it. The obtained data are in full agreement with our RNA-SEQ results.

GRIN3A (glutamate ionotropic receptor NMDA type subunit 3A).

**Figure 11:**
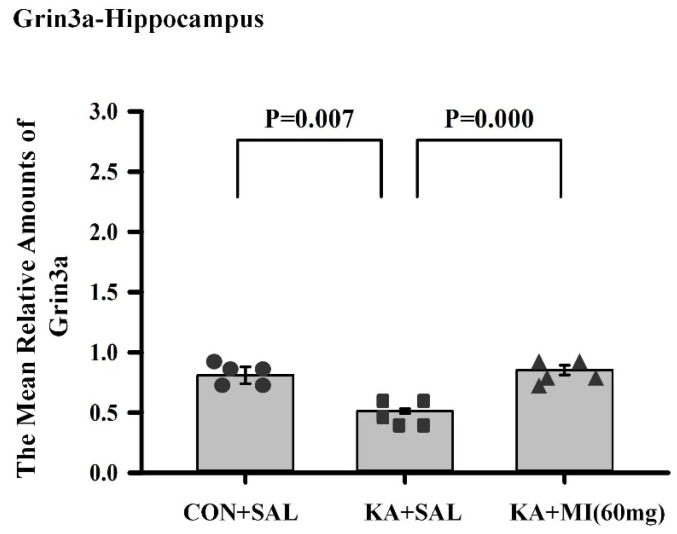
Mean relative amounts of GRIN3A mRNA in the hippocampus of CON+SAL, KA+SAL, and KA+MI 60 mg groups. Expression of GRIN3A was significantly less in the KA+SAL group as compared to both the CON+SAL and KA+MI groups. Individual values for each animal are shown on each bar.

One-way ANOVA revealed a significant effect of treatment (F2,14 = 11.91, *p* = 0.0001). The amount of GRIN3A mRNA was significantly higher in CON+SAL and KA+MI groups as compared to KA+SAL group (T = 3.58, *p* = 0.007 and T = 6.87, *p* = 0.0001, respectively, DF = 8 for both comparisons). No difference was found between CON+SAL and KA+MI groups (Figure 11). Thus, KA-induced epilepsy was followed by a decrease in GRIN3A, whereas MI treatment abolished this decrease.

In the neocortex, no significant differences are detected for either GRIK3 or GRIN3A (Supplementary Figures S3 and S4).

**Sodium channel beta subunit—SCNB4**

Biological pathways analysis indicates that sodium channel regulatory activity is decreased in the KA+SAL group but maintained in the KA+MI group at a control level. Expression of 2 genes from this pathway, namely SCB4 and Fxyd6, is not decreased but maintained in the KA+MI group (Supplementary Files S4 and S5).

The disruption of sodium channel function is one of the most commonly recognized causes of genetic epilepsy [16]. The molecular target of many antiepileptic drugs is the voltage-gated sodium channel. Drug-resistant temporal lobe epilepsy patients express less amount of SCNB4 as compared to non-epileptic subjects, and this is considered a key factor in AED-resistant TLE [17].

**Figure 12:**
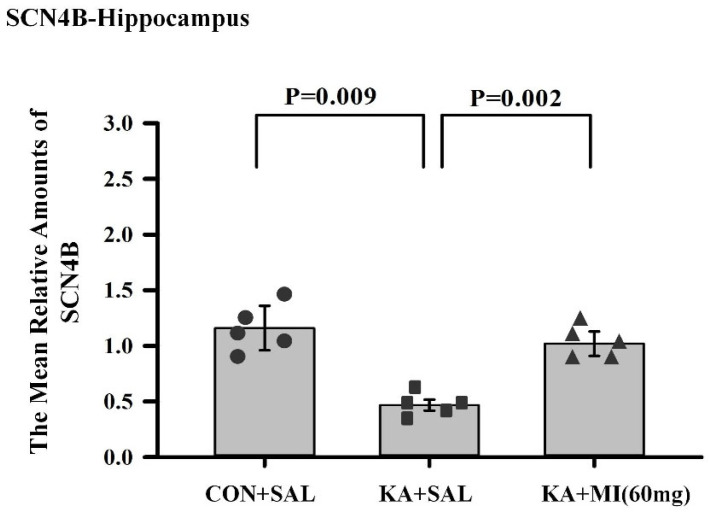
Mean relative amounts of SCNB4 mRNA in the hippocampus of CON+SAL, KA+SAL, and KA+MI 60 mg groups. Expression of SCNB4 was significantly less in the KA+SAL group as compared to both the CON+SAL and KA+MI groups. Individual values for each animal are shown on each bar.

Hippocampus. We have further validated the differential expression of SCNB4 by RT-PCR. One-way ANOVA revealed that the effect of treatment was significant (F2,14 = 7.48, *p* = 0.008). *T*-test showed that, as in the RNA-SEQ data, the expression of SCNB4 mRNA was significantly higher in CON+SAL and KA+MI groups as compared to KA+SAL group (T = 3.41, *p* = 0.009, T = 4.46, *p* = 0.002, respectively. For both comparisons, DF = 8; Figure 12). There was no significant difference between CON+SAL and KA+MI groups or between any groups in the neocortex (Supplementary Figure S5).

**Collagen 8a1**

**Hippocampus**

**Figure 13:**
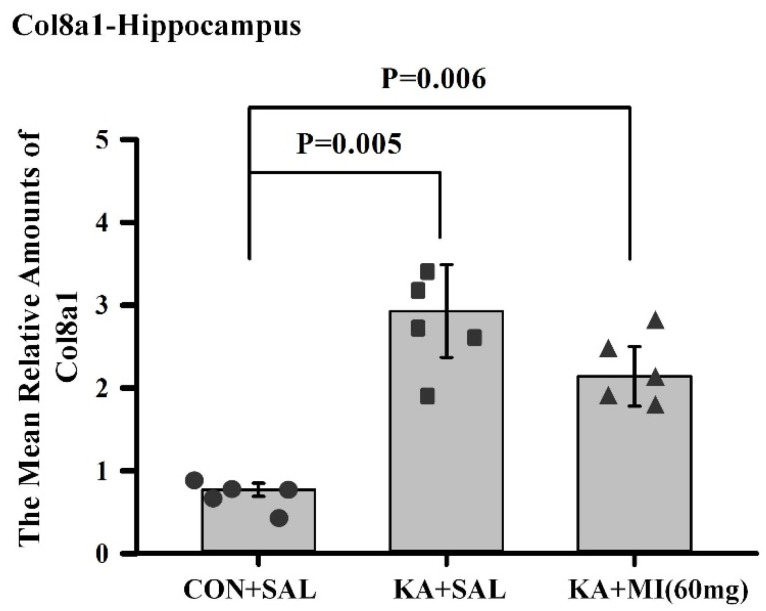
Mean relative amounts of Collagen8a1 mRNA in the hippocampus of CON+SAL, KA+SAL, and KA+MI 60 mg groups. Expression of Collagen8a1 was significantly lower in the CON+SAL group than in either the KA+SAL or the KA+MI groups. Individual values for each animal are shown on each bar.

One-way ANOVA revealed that the effect of treatment was highly significant in the hippocampus (F2,14 = 7.97, *p* = 0.007). T-test showed that the mean amounts of collagen 8a1 mRNA in KA+SAL and KA+MI groups were significantly higher than those in the CON+SAL group (T = 3.81, *p* = 0.005; T = 3.73, *p* = 0.006, respectively; DF = 8, Figure 13). There was no significant difference between the KA+SAL and KA+MI groups.

**Neocortex**

No differences were observed (Supplementary Figure S6).

**Collagen 6a1**

**Hippocampus**

**Figure 14:**
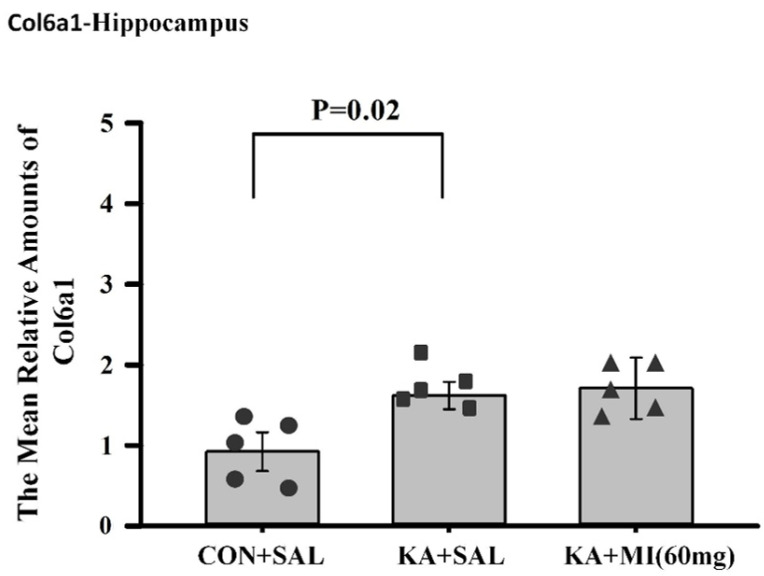
Mean relative amounts of Collagen6a1 mRNA in the hippocampus of CON+SAL, KA+SAL, and KA+MI 60 mg groups. Expression of Collagen8a1 was significantly lower in the CON+SAL group than in the KA+SAL group. Individual values for each animal are shown on each bar.

According to the RNA-SEQ data, the transcript of collagen 6a1 was significantly upregulated in the KA+SAL group as compared to CON+SAL, whereas in the KA+MI group, the upregulation was attenuated and did not reach significance (see Supplementary Tables S4 and S5). Indeed, RT-PCR confirmed significant upregulation in the KA+SAL group as compared to the CON+SAL group (one-way ANOVA, F2,13 = 4.04, *p* = 0.048; two-sample T-test T = 2.99, *p* = 0.02, DF = 8, Figure 14).

**Neocortex**

No differences were observed (Supplementary Figure S7).

**Histone Deacetylase—(HDAC7)**

The expression of this gene was significantly upregulated in the hippocampus of both KA-treated groups as compared to CON+SAL (see Supplementary Tables S4 and S5). Interestingly, the promoter region of this gene also showed altered methylation patterns, consistent with its upregulation in both KA-treated groups (see Supplementary Tables S14 and S19). In order to further validate these differences, we studied the changes in HDAC7 gene expression at the protein level.

**Hippocampus**

**Figure 15:**
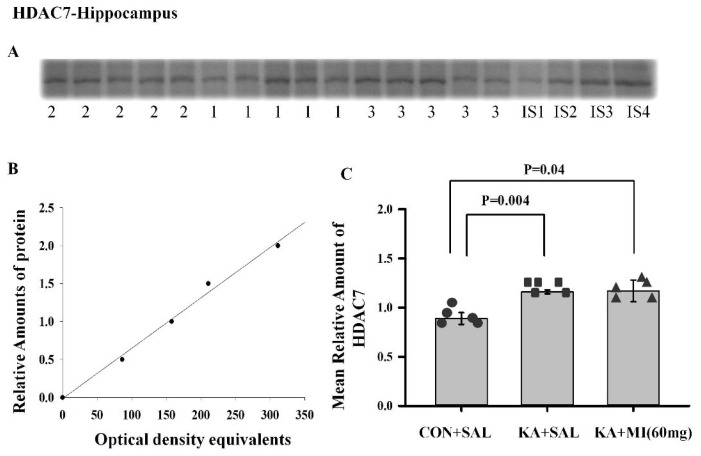
KA-induced epileptogenesis resulted in upregulation of HDAC7 in the hippocampus. (**A**) Sample film: each lane corresponds to one sample. Lanes 1–5 are from the KA+SAL group; lanes 6–10 from the CON+SAL group; and lanes 11–15 from the KA+MI group. Lanes IS-1–IS-4 are internal standards containing, respectively, 15, 30, 45, and 60 μg protein. (**B**) Calibration plot (lines fitted by linear least-squares regression). (**C**) Mean levels (mean ± standard error of the mean) of HDAC7 in hippocampus and neocortex, respectively. Individual values for each animal are shown on each bar.

One-way ANOVA revealed a significant effect of treatment (F2,14 = 5.28, *p* = 0.023). T-test showed that the HDAC7 protein level was significantly higher in both KA+SAL and KA+MI groups as compared to the CON+SAL group (T = 3.97, *p* = 0.004; T = 2.43, *p* = 0.041, respectively; DF = 8, Figure 15).

**Neocortex**

No differences were significant (Supplementary Figure S8).

The details of statistical comparisons are provided in the manuscript. Supplementary Figure S9 provides Ponceau S-stained membranes and uncropped blot images.

## 3. Discussion

Obtained data convincingly indicate dose-dependent MI effects on KA-induced epileptogenesis, specifically on (i) reductions in electrophysiological and motor SRS and the frequency of IIS; (ii) improvement of learning and memory deficits in MWM; and (iii) selected biological pathways identified in a broad range of long-term transcriptomic and DNA methylome changes associated with epilepsy. To our knowledge, this is the first study to demonstrate long-term, large-scale transcriptomic and DNA methylome alterations following MI treatment in a kainic acid-induced status epilepticus model.

**Dose-Dependence of MI effects**

We tested the effects of different MI concentrations (30 mg/kg, 60 mg/kg, and 120 mg/kg) on epileptogenesis in the KA epilepsy model in rats. We found that MI had long-lasting, dose-dependent effects on motor and electrographic manifestations of epileptogenesis. The 60 mg/kg dose was identified as the most effective, while the lower 30 mg/kg and higher 120 mg/kg concentrations displayed less efficacy. Notably, in MWM spatial learning and memory task experiments, analysis of learning dynamics indicated that the 120 mg/kg MI dose displayed the highest efficacy in mitigating the decline of spatial learning, while other KA-treated groups also displayed spatial learning, albeit to a lesser degree. Interestingly, in the spatial memory test, the 30 mg/kg and 60 mg/kg MI doses significantly alleviated spatial memory deficits, whereas the KA+MI 120 mg and KA+SAL groups did not show spatial memory. This apparent inconsistency between learning and memory tasks is probably related to the learning paradigm, which required four training trials daily over four days, while spatial memory was tested in a single trial on the fifth day.

It is important to note that in the present study, electrographic spontaneous recurrent seizures (SRS), interictal spikes (IIS), and epileptiform activity were significantly suppressed by the 60 mg/kg dose of MI, whereas the higher 120 mg/kg dose was less effective. Although the difference in suppression of motor SRS between doses did not reach statistical significance, a clear numerical trend favored the 60 mg/kg dose. This pattern was further supported by performance in the spatial memory task, where the 60 mg/kg dose also showed greater efficacy. These findings collectively suggest that the pathological hyperexcitability of the hippocampus during epileptogenesis is selectively and effectively mitigated by an MI dose near 60 mg/kg. Doses above this threshold appear to lose efficacy or even exert disruptive effects, as previously reported by Williams and Jope [14].

Interestingly, in the spatial learning task (as opposed to memory retention), the 120 mg/kg dose demonstrated high effectiveness. This apparent discrepancy may be explained by either (i) task-specific mechanisms of learning versus memory or (ii) the possibility that the optimal dose of MI for learning lies between 60 and 120 mg/kg, with efficacy tapering off at the upper end of this range.

It is noteworthy that MI exhibited a non-linear, inverted U-shaped dose–response relationship, with lower doses producing stronger antiepileptogenic effects than the highest dose (120 mg/kg). Such non-linear responses are common in biological systems, particularly in CNS receptor pharmacology [18]. Mechanistically, this may reflect the activation of multiple signaling pathways with differing sensitivity to MI concentration, resulting in selective engagement of distinct transduction mechanisms at different dose levels.

Data obtained in the current study agree with our previous report that, under a similar experimental paradigm, the MI threshold concentration of 30 mg/kg suppresses motor and electrographic SRS, molecular changes, as well as spatial learning and memory deficit comorbidities associated with TLE epileptogenesis [10]. Importantly, the 60 mg/kg MI dose not only selectively and effectively suppressed the electrographic and motor SRS frequency, but also significantly decreased the frequency of IIS—an effect not observed after treatment with the 30 mg/kg dose of MI [10]. IIS represents an electrophysiological marker of excessive increase in forebrain excitability in general and hippocampus in particular [1]. Thus, our data strongly indicate that the pathological increase in hippocampal excitability during epileptogenesis is mitigated selectively and effectively by 60 mg/kg MI. This is consistent with our hypothesis that MI helps maintain neuronal excitability within a normal range. Furthermore, it has been suggested that disruption of pyramidal neuron firing homeostasis and, consequently, excessive increase in excitability disrupts the specificity of long-term potentiation (LTP)—the proposed cellular mechanism of learning—and corrupts existing memory engrams, thus undermining declarative learning and memory [19]. The described disruption of LTP and the established synaptic weights are likely to contribute to the comorbidity of spatial memory and learning deficits associated with TLE. Thus, MI-induced mitigation of spatial learning and memory deficits observed in our experiments is consistent with the limited increase in hippocampal excitability caused by MI treatment during epileptogenesis.

**Transcriptomic changes**

Both KA+SAL and KA+MI treatments were associated with massive transcriptomic changes, not only compared with the CON+SAL group, but also between KA-treated groups. Not surprisingly, KA+MI treatment induced a larger number of gene expression changes compared with controls than did KA+SAL treatment, leading to significant transcriptomic differences between the KA+SAL and KA+MI groups. At least part of these differences should be responsible for the attenuation of KA-induced epilepsy in the KA+MI group.

There are several genes and corresponding biological pathways commonly differentially expressed in KA-treated groups relative to CON+SAL. These pathways include integrin binding, growth factor binding, transmembrane protein kinase activity, immune receptor activity, and extracellular matrix binding. The KA+MI transcriptome compared with CON+SAL also revealed differential expression of collagen binding activity—an upregulated pathway closely related to extracellular matrix binding. These pathways could be related either to epileptogenesis or to compensatory changes against it. Taking into account that epileptogenesis was clearly reduced in the MI-treated group, we suggest that increased amounts of various collagens are linked to compensatory changes against epilepsy. This suggestion is supported by recent data from Ramos-Moreno et al. [20], which showed that upregulation of Col6a1 after seizures leads to decreased glutamatergic transmission and reduced network excitability.

The most intriguing aspect of comparative RNA-SEQ data concerns the differences in sodium channel subunits and glutamate ionotropic receptor subunits. Namely, in the hippocampus of the KA+MI group, upregulation of the GRIK3 glutamate receptor subunit was observed, and its level was significantly higher compared with both KA+SAL and CON+SAL groups. In the case of SCNB4 and GRIN3A, MI treatment prevented their KA-induced downregulation, and expression remained at control levels. How can this apparent paradox—higher levels of excitatory proteins alongside markedly decreased seizure activity—be explained? We speculate that these changes are cell-type specific and propose the following scenario for MI-targeted action:

We propose that the described ion channel subunit changes primarily occur in hippocampal pyramidal neurons. It has been demonstrated in TLE rat models that during epileptogenesis and chronic epilepsy, there is downregulation of hyperpolarization-activated, cyclic nucleotide-gated (HCN) ion channels in the CA1 pyramidal neuron apical dendrite [21]. Indeed, under our experimental design, in KA+SAL animals compared with CON+SAL, there is a significant downregulation of HCN1 gene expression (see Supplementary File S5). Decreased conductance increases excitability of pyramidal neurons by raising input resistance of the apical dendrite, enhancing temporal summation of excitatory synaptic potentials in the dendrite, and promoting their spread from dendrite to soma [21]. We speculate that the upregulation of GRIK3 kainate glutamate receptor subunits in KA+MI hippocampi occurs preferentially in the CA1 pyramidal neuron apical dendrites, and thus compensates for HCN1 channel downregulation. Layer V pyramidal apical dendrites have a high density of kainate receptors, mainly extrasynaptic, activated by ambient or spillover glutamate [22]. We propose that MI treatment specifically upregulates extrasynaptic kainate receptors in these dendrites, increasing resting “leak” conductance, lowering input resistance, and decreasing both the membrane and space constants. These homeostatic effects likely lower CA1 pyramidal neuron excitability and reduce the propensity for epileptiform activity. From this follows that downregulation of GRIK3 in KA+SAL is a pro-epileptic change, increasing CA1 excitability.

GRIN3A NMDA receptor subunits are unconventional because they render NMDA receptors less calcium permeable, making synapses less prone to LTP [23]. We speculate that GRIN3A downregulation in KA+SAL pyramidal neurons is a pro-epileptic change that enhances potentiation of excitatory synapses. In KA+MI, GRIN3A remains at control levels, contributing to normal excitability.

SCNB4, the sodium channel β4 subunit, is expressed in hippocampal pyramidal neurons [24]. Its co-expression with sodium channel α-subunits shifts activation to more negative potentials [24]. We propose that downregulation of SCNB4 in KA+SAL is pro-epileptic, shifting activation positively, facilitating action potential backpropagation, and increasing excitability [25]. Restoration of SCNB4 in KA+MI likely contributes to the recovery of normal excitability.

**RNA-SEQ; Overlap with previous data**

Our earlier experiments (30 mg/kg MI) showed similar gene expression trends for SLC5A3 (sodium-myo-inositol transporter), GFAP (glial fibrillary acidic protein), and LRRC8A (volume-regulated anion channel subunit [10,11]. The current results confirm and extend these observations.

**Methylome changes**

Our data for the first time: (i) convincingly indicate long-term DNA methylation alterations in hippocampi of KA+SAL and KA+MI groups; (ii) provide a detailed map of altered methylation sites. Importantly, all differentially methylated sites were located in promoters or first exons.

KA+MI and KA+SAL groups both diverged from CON+SAL. Five genes were hypermethylated in both KA groups (Dnajc18, Hdac7, LOC102548917, Ubxn4, Rab6b). Only two genes (Them4, Ptges3l) were commonly hypomethylated. MI also induced specific methylation changes relative to KA+SAL. Given that samples were collected four weeks after MI cessation and eight weeks after SE induction, these changes are long-lasting and likely contribute to MI’s benefits. However, the overlap with RNA-SEQ was small. Genes differentially expressed and methylated included: KA+MI vs. CON+SAL (Zfp717, Ptges3l, Hdac7); KA+SAL vs. CON+SAL (Smurf2, Arhgap24, Hdac7). None showed strict inverse methylation–expression correlation, consistent with evidence that disease-associated hypermethylation can both up- and down-regulate expression [26].

Of note, HDAC7 was upregulated in both KA groups at RNA and protein levels, with hypermethylation also detected. HDACs generally repress transcription via histone deacetylation [27] but also mediate non-canonical functions, e.g., neuroprotection [28]. HDAC7 downregulation is linked to depressive behavior [29]. Our earlier work showed MI mitigates apoptosis after SE [30], potentially via HDAC7.

**LncRNAs and KA-induced SE**

Among differentially expressed transcripts (KA+SAL and KA+MI vs. CON+SAL), 15 were lncRNAs (Supplementary Tables S4 and S5). ENSRNOG00000064277 was upregulated only in the hippocampus, enriched in the cytoplasm, not the nucleus. LncRNAs regulate chromatin, methylation, R-loops, and splicing [31] but also act in cytoplasm—for instance, glycoLINC scaffolds glycolytic metabolons [32]. LncRNAs are increasingly implicated in epilepsy [33,34]. Thus, lncRNAs identified here merit further study as therapeutic targets.

It should be emphasized that the observed transcriptomic and DNA methylation alterations following MI and kainic acid treatment are associative and may not directly account for the behavioral or electrographic antiepileptogenic effects.

## 4. Materials and Methods

### 4.1. Animals

Animals were housed individually and maintained under a 12 h light/12 h dark cycle and had free access to food and water. Experimental design was approved by the Bioethics Committee of the I. Beritashvili Centre of Experimental Biomedicine (Protocol N03/1 November 2019). We monitored health parameters—body weight and mortality of the animals. These parameters stayed normal across animal groups and were not different between the groups.

### 4.2. KA-Induced SE

Male Wistar rats, 2.5–3 months of age, received a single intraperitoneal (IP) injection of kainic acid (KA; 10 mg/kg, Abcam, Cambridge, UK, Cat. No. 120100) dissolved in saline. After injection, each animal was placed in an individual plastic cage for observation for 4 h. Seizures were scored according to a modified Racine scale (0–6) [35,36].

The seizure intensity (scores) and durations on video recordings were measured by two coauthors (not aware of the injection status), substituting for each other for a weekly period. Thus, the data regarding motor seizures were obtained in a blind manner.

For further experiments, only animals that exhibited a 4–6 score SRS and with a total duration of at least 60 min during the 4 h observation period were selected. This selection was used to ensure induction of epileptogenesis and, consequently, the development of SRS (i.e., epilepsy). In each series of experiments, selected KA-treated rats were injected with different doses of MI, 30 mg/kg, 60 mg/kg, or 120 mg/kg, and designated as KA+MI30, KA+MI60, and KA+MI120 groups, respectively. One KA-treated group received intraperitoneal (IP) saline (0.9% NaCl sterile solution, 1 mL/kg) instead of MI and was designated as KA+SAL.

IP injections (twice daily) of MI or saline started 4 h following KA treatment and continued for 28 days after the start of the experiment. The control group of animals (CON+SAL), healthy intact animals, received, at exactly the same time schedule, an intraperitoneal injection of only saline (0.9% NaCl solution, 1 mL/kg) twice daily for 28 days. To observe long-term effects of MI, after termination of MI or saline injections, animals were maintained in cages without treatment for an additional 4 weeks. The animals were continuously video-monitored (24 h) in their cages. At the end of 8 weeks from the injection of KA, EEG was recorded in KA-treated animals. Finally, after the termination of the EEG sessions, the animals were tested in the Morris Water Maze (MWM).

**Figure 16:**
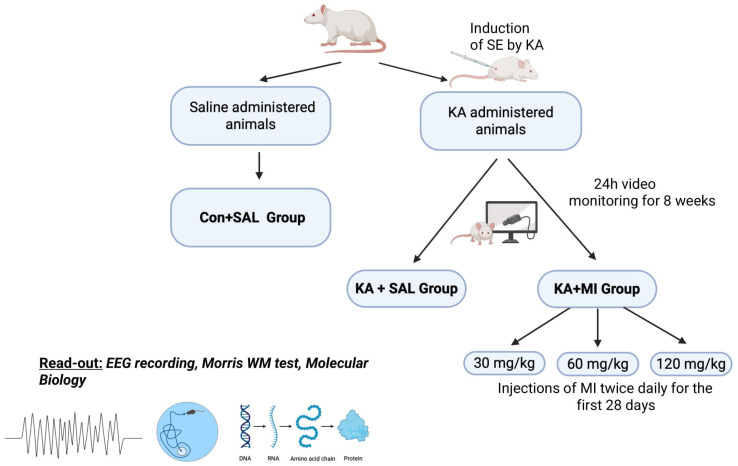
Schematic depiction of the experimental design (details are provided in the manuscript).

The most effective MI dose identified in the behavioral and electrographic experiments was used in epigenetic and transcriptome studies (Figure 16).

### 4.3. Video-Monitoring

Animals were housed individually and maintained under a 12 h light/12 h dark cycle. Animal behavior was monitored continuously (24/7) by infrared video cameras (IRIP66 HIK VISION, Hangzhou, China) as described in our previous paper [10]. Only seizures of grades 4–6 were identified and evaluated, since lower seizure grades can easily be confused with normal behavior.

The number of animals for motor seizure analyses in different KA-treated groups was as follows: KA+SAL n = 14, KA+MI (30 mg) n = 10, KA+MI (60 mg) n = 11, and KA+MI (120 mg) n = 11.

### 4.4. Surgery and EEG Recording

At 28 days after termination of treatment with different doses of MI or with saline, a steel bipolar electrode was implanted in the right dorsal hippocampus of KA-injected animals under ketamine/xylazine anesthesia using the rat brain atlas [37]. The following coordinates were selected for the placement of the electrode in the right hippocampus: 4.5 mm caudally from the bregma, 2.8 mm laterally from the midline, and 3 mm ventral to the skull surface. The animals were allowed to recover for 2 days after surgery, and hippocampal EEG was recorded for the following 3 days, 3 h per day.

Recording periods were alternated in the morning and afternoon between KA+SAL and KA+MI rats to ensure that each group was recorded for equivalent periods of the day.

An EEG was recorded with an amplifier (Pinnacle Technology, Ottawa Lake, WI, USA), low-pass filtered at 50 Hz, and acquired with the software Sirenia version 2.2.7 (Pinnacle Technology, Ottawa Lake, WI, USA). The duration, frequency, and number of electrographic SRS were analyzed.

The SRS activity was identified as high-frequency spike-and-wave activity with the amplitude exceeding the background activity at least twice. Additionally, the number of interictal spikes was evaluated. Interictal spikes were identified as brief, sharp negative or negative-positive deflections with a duration < 1 s and an amplitude at least twice that of the background.

In different KA-treated groups, the number of animals used for EEG analyses was as follows: KA+SAL n = 6, KA+MI (30 mg) n = 8, KA+MI (60 mg) n = 8, and KA+MI (120 mg) n = 7.

### 4.5. Morris Water Maze (MWM)

MWM test [38] was used to study spatial learning and memory in the following groups of animals: KA+SAL, KA+MI 30 mg, KA+MI 60 mg, KA+MI 120 mg, and CON+SAL. The MWM test was conducted on the same animals used in electrophysiological studies (see above). Animal training/testing was carried out as described in our previous paper [10], with a 4-day learning session and spatial memory testing on the 5th day. The number of animals in different KA-treated and control groups used for the MWM test was as follows: CON+SAL n = 10, KA+SAL n = 7, KA+MI (30 mg) n = 5, KA+MI (60 mg) n = 7, and KA+MI (120 mg) n = 8. The training protocol for different groups of animals was identical.

### 4.6. Molecular Biological Studies

The optimal dose of MI identified in behavioral and electrographic experiments was utilized in molecular biology studies. To this end, transcriptomics (RNA-seq) and methylome (reduced representation bisulfite sequencing, RRBS-seq) experiments were performed on rats’ hippocampus with the design described above (Figure 16). Each experiment included CON+SAL, KA+SAL, and KA+MI (60 mg/kg) groups of rats, each group containing 4 animals. At the end of eight weeks from KA injection, rats were decapitated, and the hippocampus was removed from each brain and immediately frozen on dry ice. The hippocampus samples were shipped on dry ice to the commercial service provider Diagenode, for carrying out both types of experiments (see subsections below for details).

The validation of RNA-seq results was carried out using real-time polymerase chain reaction (RT-PCR) assay and Western immunoblotting on hippocampus and neocortex samples. Each group of animals contained 5 rats.

#### 4.6.1. RNA-SEQ

The RNA-SEQ experiment on 12 hippocampal samples (4 samples from each CON+SAL, KA+SAL and KA+MI (60 mg/kg) groups) was performed by RNA-SEQ services (Diagenode, Seraing, Belgium, Cat# G02030000). RNAs were extracted using the RNeasy Mini Kit (Qiagen, Venlo, The Netherlands, #74104). RNA was quantified using the Qubit™ RNA BR Assay Kit (Thermo Fisher Scientific, Waltham, MA, USA, Q10210) and further assessed for integrity using the RNA 6000 Pico Kit (5067-1513, Agilent, Santa Clara, CA, USA) on a 2100 Bioanalyzer system (Agilent, Santa Clara, CA, USA).

The 12 RNA samples were processed together, and library preparation was performed with 500 ng of input RNA using: NEBNext^®^ rRNA Depletion Kit (Human/Mouse/Rat) (NEB #E6310), followed by NEBNext Ultra II Directional RNA Library Prep Kit for Illumina (NEB #E7760) and NEBNext^®^ Multiplex Oligos for Illumina^®^ Index Primers Set 1 (NEB #E6440). The other details of the experiments were analogous to those described in our previous publication [39]. Illumina sequencing was applied with paired-end 50 bp reads, generating on average 50 M raw reads/sample (Diagenode G02030003).

#### 4.6.2. Reduced-Representation Bisulfite Sequencing (RRBS)

The RRBS experiment was conducted on 12 hippocampal samples (4 samples from each of CON+SAL, KA+SAL, and KA+MI groups). DNA Methylation Profiling (RRBS Service, Diagenode Cat# G02020000) was performed for this purpose. Genomic DNA quality control, RNase treatment, RRBS library preparation, library quality control, and deep sequencing were conducted according to methods described in our previous paper [39] at Diagenode, and details are provided in Supplementary File S21.

#### 4.6.3. RT-PCR

**RNA Isolation.** RNA was isolated separately from the hippocampus and neocortex using the NucleoSpin RNA Total RNA Isolation Kit (Macherey-Nagel, Düren, Germany, Cat. No. 740955.50).

The concentration of RNA was measured by absorbance at wavelengths 280/260 nm on a Nanodrop.

**mRNA Measurement.** Complementary DNA (cDNA) from the RNA fractions of hippocampus and neocortex was synthesized using the High-Capacity cDNA Reverse Transcription Kit (Thermo Fisher Scientific, Cat. No. 4368814). Relative cDNA copy number was determined by real-time PCR using the Step One Real-Time PCR System (Applied Biosystems, Waltham, MA, USA) with the SYBR Green detection method and was normalized to beta-actin. The sequences of primers for selected genes are provided in Supplementary File S22 (Supplementary Table S21).

#### 4.6.4. Electrophoresis and Immunoblotting

Protein Determination, Electrophoresis, and Western Immunoblotting were carried out according to previously described methods [9]. After the electrophoresis, the proteins were transferred onto nitrocellulose membranes (0.45 microns) and stained with Ponceau S solution (0.1%), digitalized, and uniform gel loading and transfer were confirmed with Lab Works 4.0 (UVP). HDAC-7 anti-HDAC7 primary antibody (ABIN7263279, Antibodies Online) was used for the detection of the target protein with established immunochemical procedures [9].

The optical densities of the bands corresponding to HDAC-7 were measured using Lab Works 4.0 (UVP). The autoradiographs were calibrated by including in each gel 4 standards of homogenate protein fraction (15, 30, 45, and 60 μg of corresponding total protein) obtained from the control rats. The optical density of each sample band was divided by the optical density of the band for the 30 μg protein standard to give the “relative amount of protein”, and data obtained in such a way was used for statistical analysis. Data were not normalized to actin or any housekeeping protein in brain tissue samples, because such proteins cannot be guaranteed to remain unchanged by KA-induced SE. We controlled gel loading by Ponceau S staining, Lab Works 4.0 (UVP) analysis, and calibration with protein standards

### 4.7. Statistical Methods

#### 4.7.1. Electrographic SRS Frequency, Electrographic SRS Duration, Motor SRS Frequency, and Motor SRS Duration

A one-way analysis of variance (ANOVA) and Tukey’s HSD (honestly significant difference) test were used to assess differences among groups for electrographic SRS frequency and duration. For motor SRS frequency and duration, one-way ANOVA and two-sample *t*-tests were applied.

#### 4.7.2. Interictal Spikes

The Shapiro–Wilk test was used to test the hypothesis of normal distribution. The Kruskal–Wallis test, as well as the Wilcoxon rank-sum test with the Holm correction, were used to assess the significance of differences in a non-parametric way.

#### 4.7.3. Spatial Learning and Spatial Memory Task

A two-way analysis of variance (ANOVA) and Tukey’s HSD test were used to assess differences across two factors. Finally, two-sample *t*-tests were used to assess differences in escape latent period on the 4th day between the control (CON+SAL) and the other groups.

#### 4.7.4. Bioinformatics Analysis

**Preprocessing of RNA-seq data**

The raw fastq files were analyzed with the nf-core/rnaseq 3.11.2 pipeline [40], with default parameters and using the mRatBN7.2.110 Ensembl genome reference [41]. This pipeline first performs quality control with FastQC, followed by adapter and quality trimming with Trim Galore, and then quantifies gene expression count matrices with STAR [42] and Salmon [43].

**Preprocessing of methylation (bisulfite) sequencing data**

Methyl-seq data were analyzed with the nf-core/methylseq 2.6.0 pipeline [40], using default settings, and the same genome reference as for the RNA-seq data (mRatBN7.2.110). This pipeline uses FastQC and Trim Galore! for quality control and read trimming, followed by alignment and methylation level quantification using the Bismark suite [44].

**Analysis of RNA-seq data**

For each contrast, the DESeq2 R package, (version 1.44) [45] was used to identify differentially expressed genes from the count matrix. In short, DESeq2 fits a generalized linear model for each gene, which includes a term representing the contrast of interest. The statistical significance of this term was used to assess whether the gene is differentially expressed. Furthermore, DESeq2 uses a shrinkage approach to produce robust estimates of log fold change and variances.

Enrichment of differentially expressed genes in Gene Ontology [46] biological processes were performed with a hypergeometric test, as implemented in the package clusterProfiler (version 4.12) [47], considering genes with adjusted *p*-value ≤ 0.05 as differentially expressed.

**Analysis of methylation (bisulfite) sequencing data**

For each contrast, differentially methylated sites were identified with the limma R package, (version3.60) [48]. In a similar fashion to DESeq2, limma fits a linear model for each methylation site, including a term representing the contrast of interest. The statistical significance of this term was used to assess whether the site is differentially methylated. A shrinkage approach was applied to produce robust variance estimates. Enrichment analysis was performed as described for the RNA-seq data.

All *p*-values were adjusted for multiple testing with the false discovery rate (FDR) approach [49] in both analyses.

## 5. Conclusions

Our results reveal long-term molecular targets of MI treatment in KA-induced epilepsy. However, early cellular targets remain unclear. We propose three direct mechanisms of MI action [10,15]: (i) osmotic stabilization after seizures, (ii) modulation of phosphoinositide signaling, augmenting muscarinic K^+^ currents and reducing excitability [50], (iii) modulation of GABA-A receptor function [6].

In conclusion, MI suppressed epileptogenesis in KA rats in a dose-dependent manner. Interestingly, the intermediate dose (60 mg/kg) was most effective, suggesting that MI’s protective effect requires balanced activation of signaling and gene expression mechanisms. Data strongly suggest MI limits hippocampal pyramidal neuron hyperexcitability, via membrane, synaptic, and network homeostasis mechanisms. Our work, therefore, highlights MI as a promising anti-epileptogenic drug. In support of our suggestion, recent clinical studies have shown that enteral MI therapy in a patient with genetic epilepsy is associated with improvement in seizure burden and stabilization [51].

## 6. Strengths, Limitations, and Future Directions

The main strengths of this study are as follows: (i) identification of an optimal MI concentration that attenuates epileptogenesis; (ii) characterization of gene expression and DNA methylation changes following kainic acid–induced epilepsy and MI treatment eight weeks after initial status epilepticus; and (iii) delineation of molecular pathways associated with MI’s effects.

The principal limitation is that RNA-seq was conducted on whole hippocampal tissue, limiting cell-type resolution (e.g., principal neurons vs. interneurons).

Future work will apply single-cell RNA-seq and interventional approaches to dissect the specific cellular and molecular mechanisms underlying MI’s antiepileptogenic effects.

## Data Availability

The original contributions presented in this study are included in the article/Supplementary Material. Further inquiries can be directed to the corresponding authors.

## Supplementary Materials

The following supporting information can be downloaded at: https://www.mdpi.com/article/10.3390/ijms262211102/s1.
