# Supplementary material for: Dose-Dependent Effects of Myo-Inositol on Kainic Acid-Induced Epilepsy: Electrophysiological, Behavioral, Transcriptomic, and DNA Methylome Studies
Source: Int J Mol Sci. 2025 Nov 17;26(22):11102. doi: 10.3390/ijms262211102 (PMC12652981; doi:10.3390/ijms262211102)
Supplement: Supplementary file 1 [file ijms-26-11102-s001.zip › Supplementary File_S1.pdf]

Supplementary Table S1. Data of statistical comparison between the different group of rats for:(A) SRS frequencies and (B) SRS durations. Data only for significant differences are provided.

**A -SRS FREQUENCY**

| Comparison between groups | P | T-value | Degrees of Freedom |
|---|---|---|---|
| SRS Frequency (8 weeks period) | | | |
| KA+SAL vs KA+MI (30mg/kg) | 0.001 | 3.64 | 22 |
| KA+SAL vs KA+MI (60mg/kg) | 0.0001 | 4.83 | 23 |
| KA+SAL vs KA+MI (120mg/kg) | 0.0001 | 4.91 | 23 |
| SRS Frequency (I-IV weeks period) | | | |
| KA+SAL vs KA+MI (30mg/kg) | 0.02 | 2.46 | 22 |
| KA+SAL vs KA+MI (60mg/kg) | 0.001 | 3.61 | 23 |
| KA+SAL vs KA+MI (120mg/kg) | 0.0001 | 4.18 | 23 |
| SRS Frequency (V-VIII weeks period) | | | |
| KA+SAL vs KA+MI (30mg/kg) | 0.001 | 3.97 | 22 |
| KA+SAL vs KA+MI (60mg/kg) | 0.0001 | 4.81 | 23 |
| KA+SAL vs KA+MI (120mg/kg) | 0.0001 | 4.65 | 23 |
| **B SRS Duration** | | | |
| **SRS Duration (8 weeks period)** | | | |
| KA+SAL vs KA+MI (30mg/kg) | 0.001 | 3.83 | 22 |
| KA+SAL vs KA+MI (60mg/kg) | 0.0001 | 4.78 | 23 |
| KA+SAL vs KA+MI (120mg/kg) | 0.0001 | 4.64 | 23 |
| SRS Duration (I-IV weeks) | | | |
| KA+SAL vs KA+MI (30mg/kg) | 0.003 | 3.09 | 22 |
| KA+SAL vs KA+MI (60mg/kg) | 0.001 | 3.95 | 23 |
| KA+SAL vs KA+MI (120mg/kg) | 0.001 | 3.96 | 23 |
| SRS Duration (V-VIII weeks) | | | |
| KA+SAL vs KA+MI (30mg/kg) | 0.001 | 3.74 | 22 |

| | | | |
|---|---|---|---|
| KA+SAL vs KA+MI (60mg/kg) | 0.0001 | 4.55 | 23 |
| KA+SAL vs KA+MI (120mg/kg) | 0.001 | 3.99 | 23 |
