# Supplementary material for: Dose-Dependent Effects of Myo-Inositol on Kainic Acid-Induced Epilepsy: Electrophysiological, Behavioral, Transcriptomic, and DNA Methylome Studies
Source: Int J Mol Sci. 2025 Nov 17;26(22):11102. doi: 10.3390/ijms262211102 (PMC12652981; doi:10.3390/ijms262211102)
Supplement: Supplementary file 1 [file ijms-26-11102-s001.zip › Supplementary File_S2.pdf]

Supplementary Table -S2A. Difference by escape latency time (seconds) between days I and IV in different group of rats

Treatment type	Difference (sec)	T-values	DF	P	Adjusted P values
CON+SAL	21.47	5.048	9	0.000346	0.00173
KA+SAL	15.4	2.503	7	0.020378	0.033963
KA+MI (30mg/kg)	22.14	1.938992	4	0.062261	0.062261
KA+MI (60mg/kg)	12.64	2.117902	6	0.039255	0.049069
KA+MI (120mg/kg)	15.125	3.264226	7	0.006892	0.01723

Supplementary Table-S2B. Difference by the time spent in quadrants I and IV (seconds) separated per treatment group

Treatment type	Difference (sec)	T-values	DF	P	Adjusted P values
CON+SAL	7.37	2.879	9	0.009	0.03
KA+SAL	-5.88	-1.68	6	0.92	0.92
KA+MI (30mg/kg)	5.44	2.71	4	0.026	0.044
KA+MI (60mg/kg)	7.92	2.99	6	0.012	0.03
KA+MI (120mg/kg)	3.64	0.98	7	0.17	0.22
