# Supplementary material for: Dose-Dependent Effects of Myo-Inositol on Kainic Acid-Induced Epilepsy: Electrophysiological, Behavioral, Transcriptomic, and DNA Methylome Studies
Source: Int J Mol Sci. 2025 Nov 17;26(22):11102. doi: 10.3390/ijms262211102 (PMC12652981; doi:10.3390/ijms262211102)
Supplement: Supplementary file 1 [file ijms-26-11102-s001.zip › Supplementary File_S21- RRBS experiments.pdf]

Reduced-representation bisulfite sequencing (RRBS)

The RRBS experiment was conducted on 12 hippocampal samples (4 samples from each of CON+SAL, KA+SAL and KA+MI groups). DNA Methylation Profiling (RRBS Service) from Diagenode (Cat# G02020000) was applied for this purpose.

Genomic DNA quality control

Genomic DNA was isolated with QIAamp DNA mini kit (Qiagen). DNA concentration of the brain samples was measured using the Qubit® dsDNA HS Assay Kit (Thermo Fisher Scientific) and DNA quality of the samples was assessed with the Fragment Analyzer TM and the DNF-488 High Sensitivity genomic DNA Analysis Kit (Agilent).

RNase treatment

Genomic DNA from 9 selected samples was treated with RNase cocktail (Thermo Fisher Scientific, AMM2288) for 30 min at 37°C to remove contaminating small RNAs. Following the treatment, DNA was purified using Agencourt AmpureXP beads (Beckman Coulter). DNA was eluted in Elution Buffer (10mM Tris-Cl, pH 8.5) and quality control steps were performed again as described above.

RRBS library preparation

The RRBS library preparation was performed by DNA Methylation Profiling Service (RRBS Service) (Diagenode Cat# G02020000). The Premium Reduced Representation Bisulfite Sequencing (RRBS) v2 Kit (Diagenode Cat# C02030036) was used for RRBS libraries preparation. 100 ng of genomic DNA were used to start library preparation for each sample.. PCR clean-up after the final library amplification was performed using a 1.45x beads:sample ratio of Agencourt® AMPure® XP (Beckman Coulter).

RRBS library pools quality control

DNA concentration of the pools was measured using the Qubit® dsDNA HS Assay Kit (Thermo Fisher Scientific). The profile of the pools was checked using the DNF-474 NGS fragment kit on a Fragment Analyzer (Agilent).

In the event of adapter dimer peaks being too high, the pools were size-selected one more time using a 1.45x beads:sample ratio of Agencourt® AMPure® XP (Beckman Coulter) and quality control steps were performed again.

Deep Sequencing

Obtained RRBS library pools were sequenced on a NovaSeq6000 (Illumina) using 50 bp paired-end read sequencing (PE50).
