# Supplementary material for: Dose-Dependent Effects of Myo-Inositol on Kainic Acid-Induced Epilepsy: Electrophysiological, Behavioral, Transcriptomic, and DNA Methylome Studies
Source: Int J Mol Sci. 2025 Nov 17;26(22):11102. doi: 10.3390/ijms262211102 (PMC12652981; doi:10.3390/ijms262211102)
Supplement: Supplementary file 1 [file ijms-26-11102-s001.zip › Supplementary File_S22.pdf]

Supplementary Table S21

Gene Name	Forward Primer	Reverse Primer
ENSRNOG00000064277-Long non coding RNA	GATGTCTTCCCTGGTGGTTATG	CTGCCTTGATTGCTGTGTTTG
Col6a1	CCCACTGACCAAACAGGAATAG	CAGACGGAACACAGACAGAAA
Col8a1	CCCATATCAAGACAAGGAGGAG	GTGGTATCTGAGGAGGGATTTG
Scn4b	CTCTACTTCAGGTGGTCCTACA	GACAGGACTCGGCATCAATATC
Grik3	GAGGCTCAATGGGAAGGATTA	GTCAGAGAGTCGGTGACATTAG
Grin3a	CCTACCCTACAACCTGTCTTTG	CCAAGTCCAGCTCCATCATT
Beta Actin	GAGATTACTGCCCTGGCTCCTA	CTGCTTGCTGATCCACATCTG
