# Supplementary figures and images for: Dose-Dependent Effects of Myo-Inositol on Kainic Acid-Induced Epilepsy: Electrophysiological, Behavioral, Transcriptomic, and DNA Methylome Studies
Source: Int J Mol Sci. 2025 Nov 17;26(22):11102. doi: 10.3390/ijms262211102 (PMC12652981; doi:10.3390/ijms262211102)

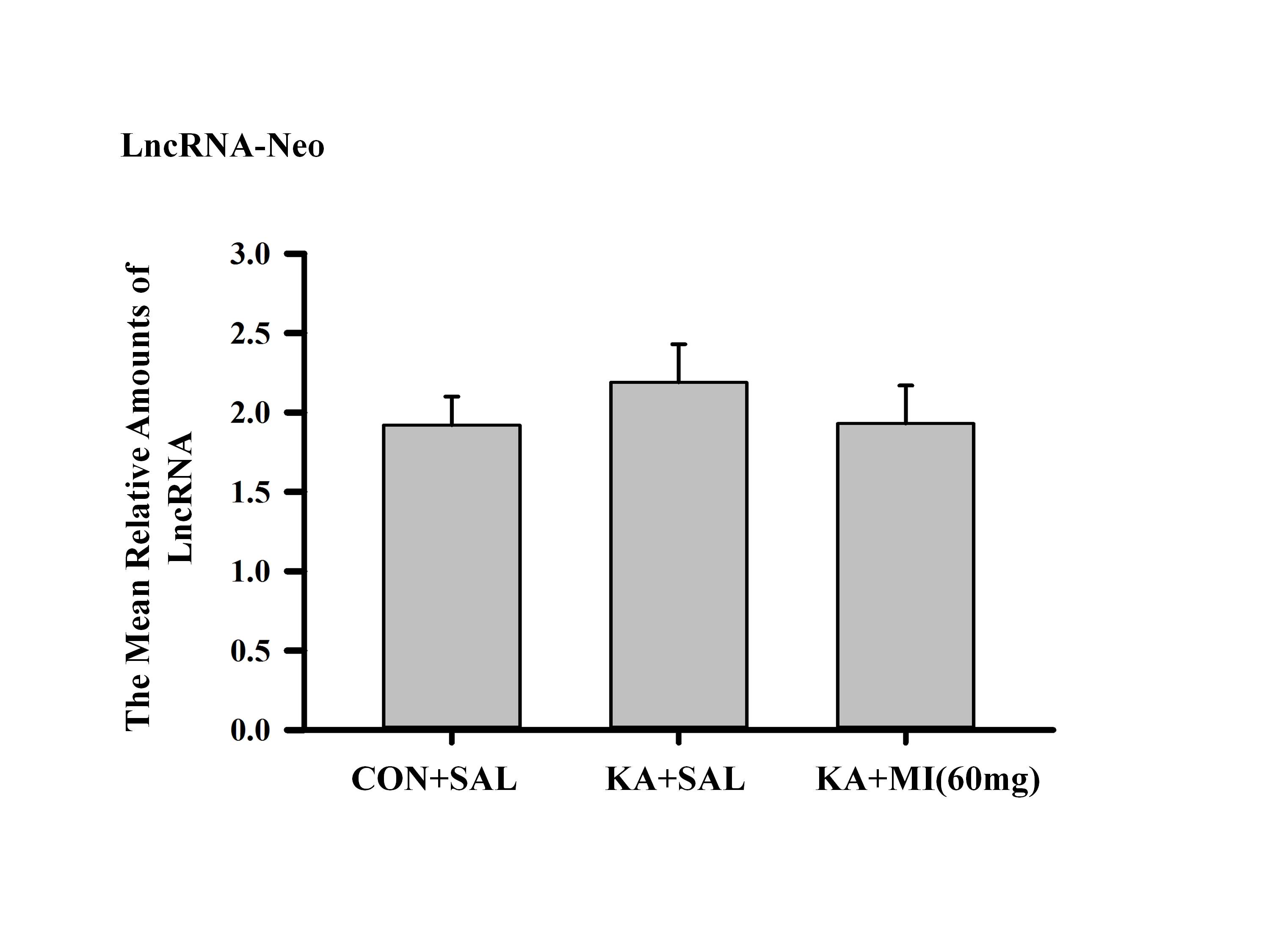

Supplement: Supplementary file 1 [file ijms-26-11102-s001.zip › Supplementary Figure S1.jpg]

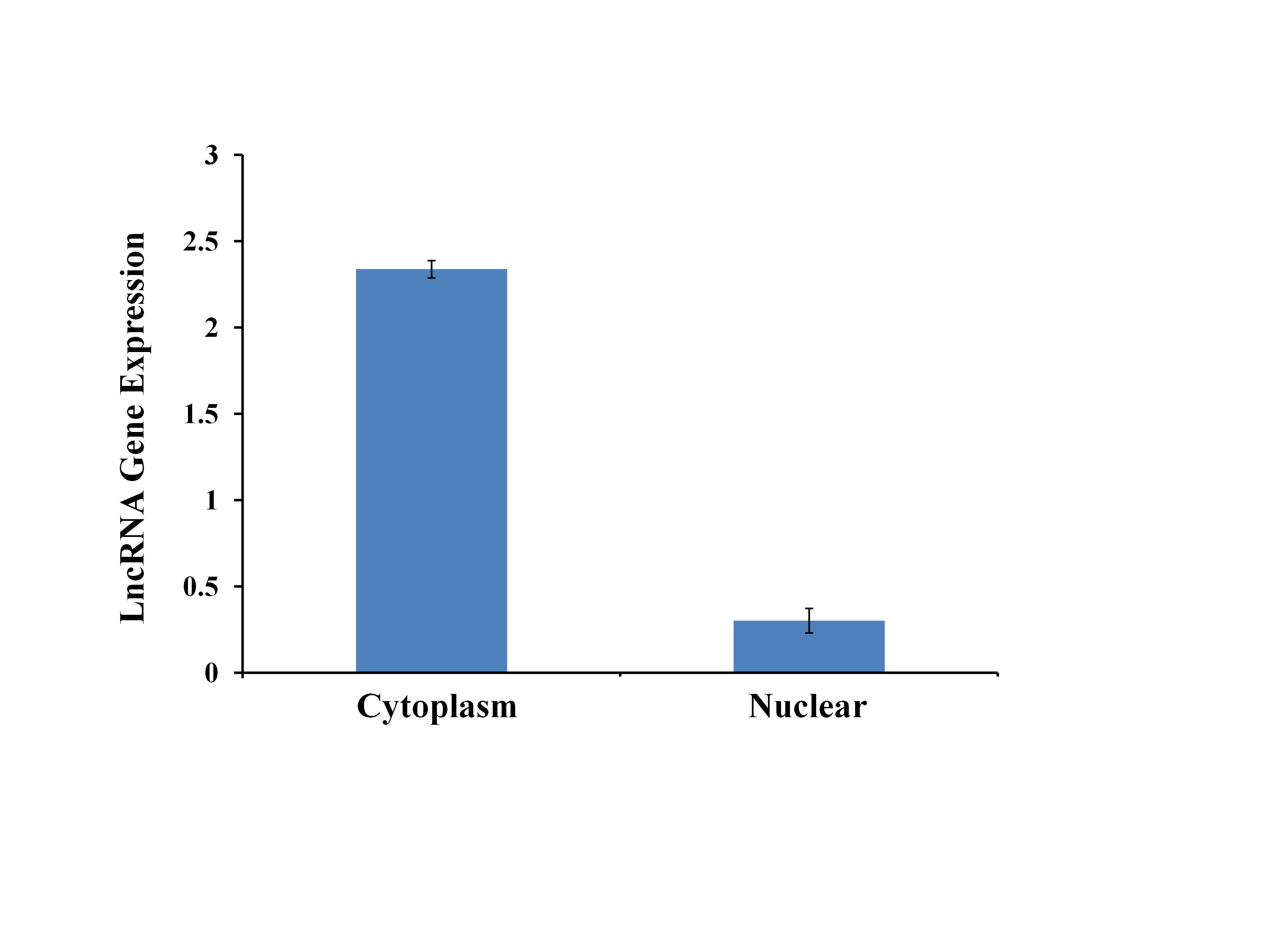

Supplement: Supplementary file 1 [file ijms-26-11102-s001.zip › Supplementary Figure S2.jpg]

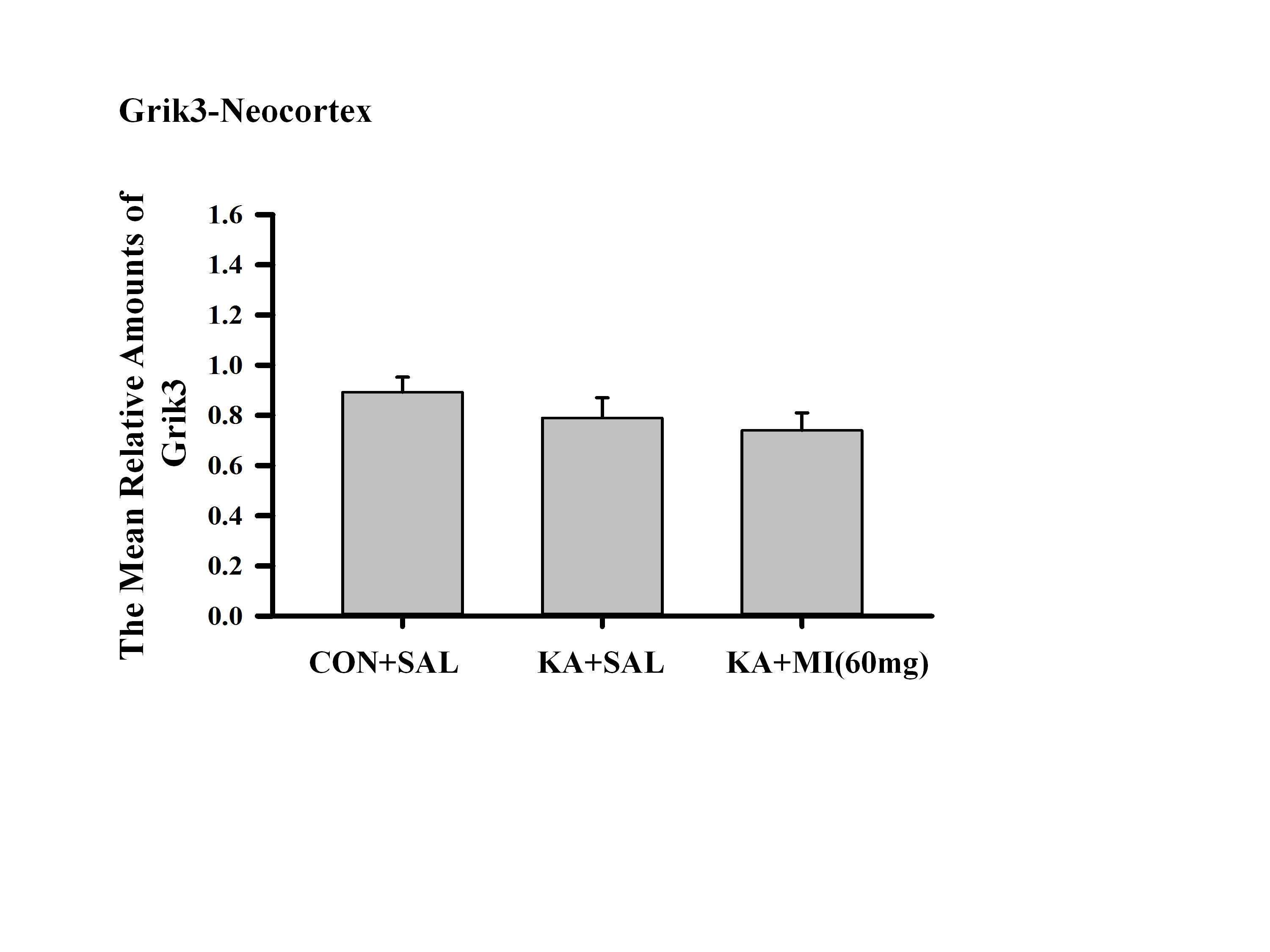

Supplement: Supplementary file 1 [file ijms-26-11102-s001.zip › Supplementary Figure S3.jpg]

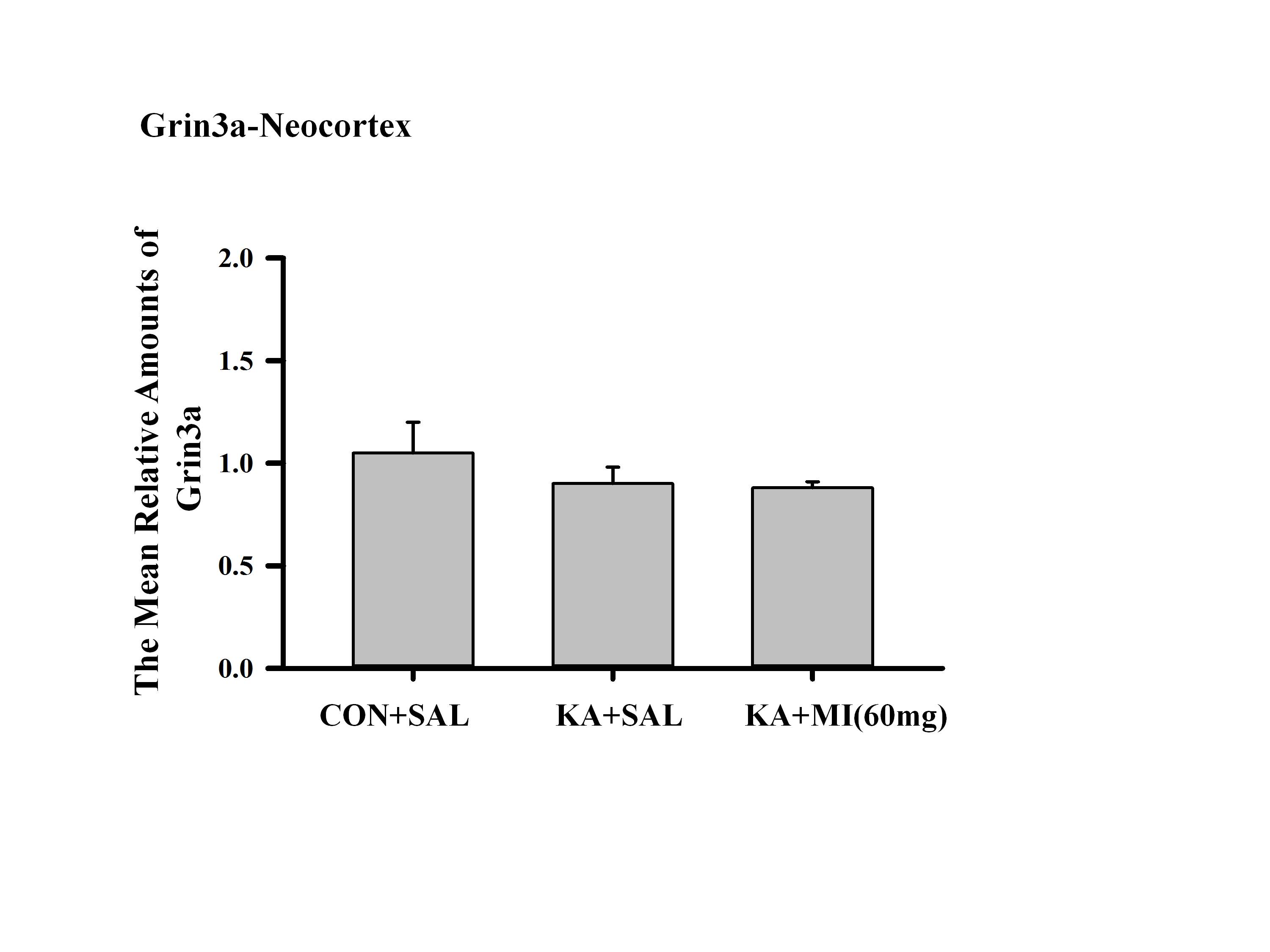

Supplement: Supplementary file 1 [file ijms-26-11102-s001.zip › Supplementary Figure S4.jpg]

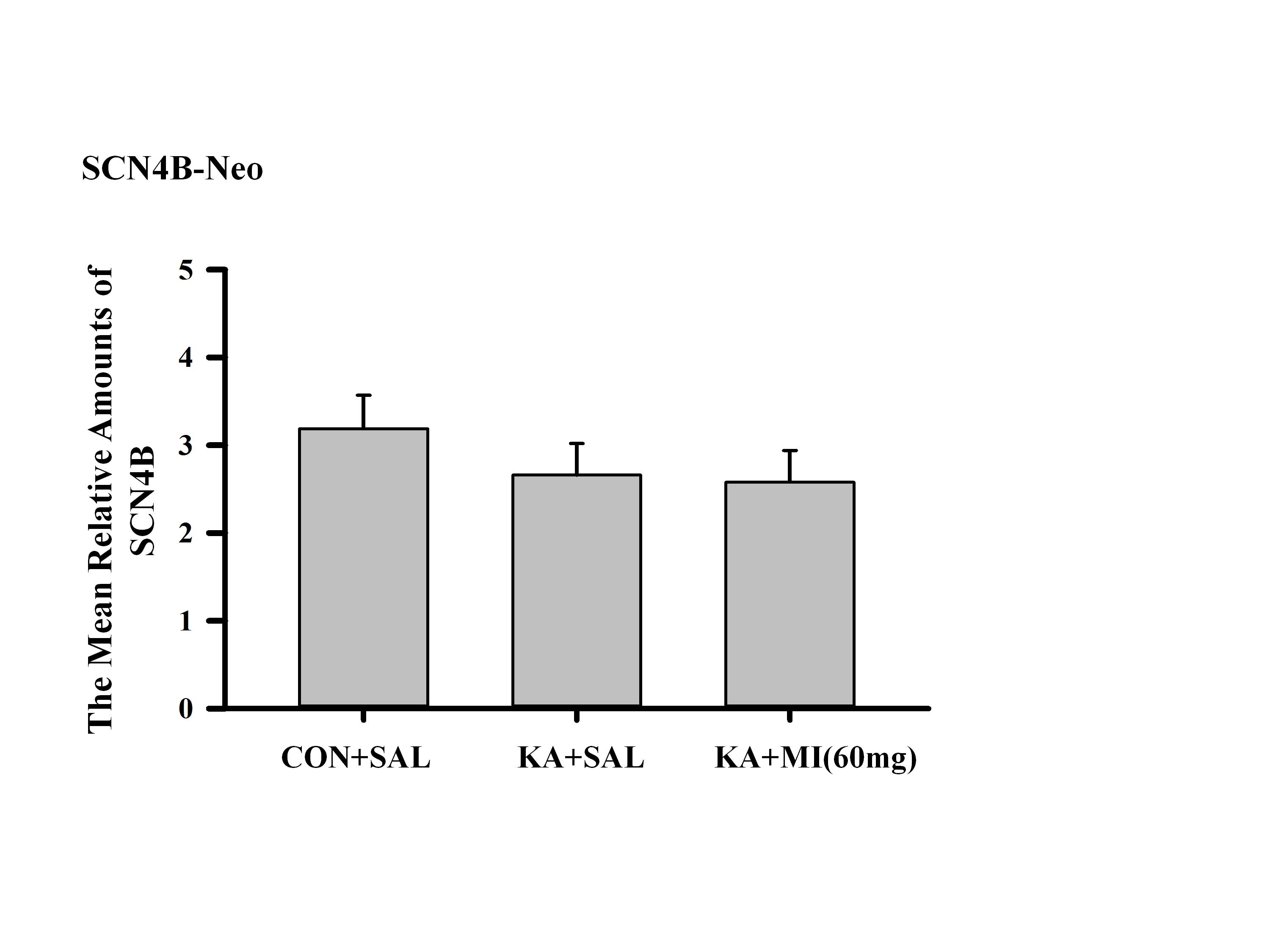

Supplement: Supplementary file 1 [file ijms-26-11102-s001.zip › Supplementary Figure S5.jpg]

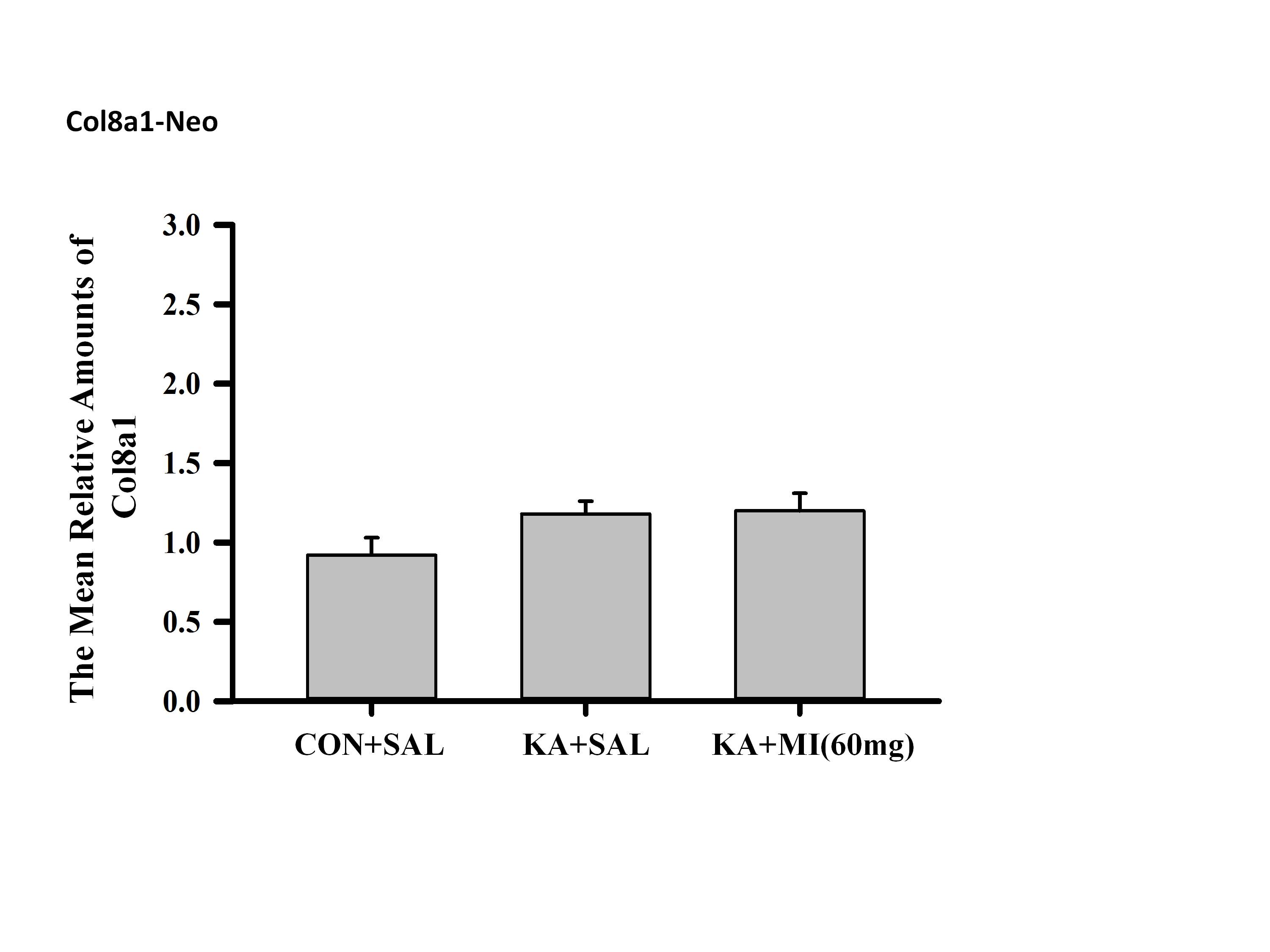

Supplement: Supplementary file 1 [file ijms-26-11102-s001.zip › Supplementary Figure S6.jpg]

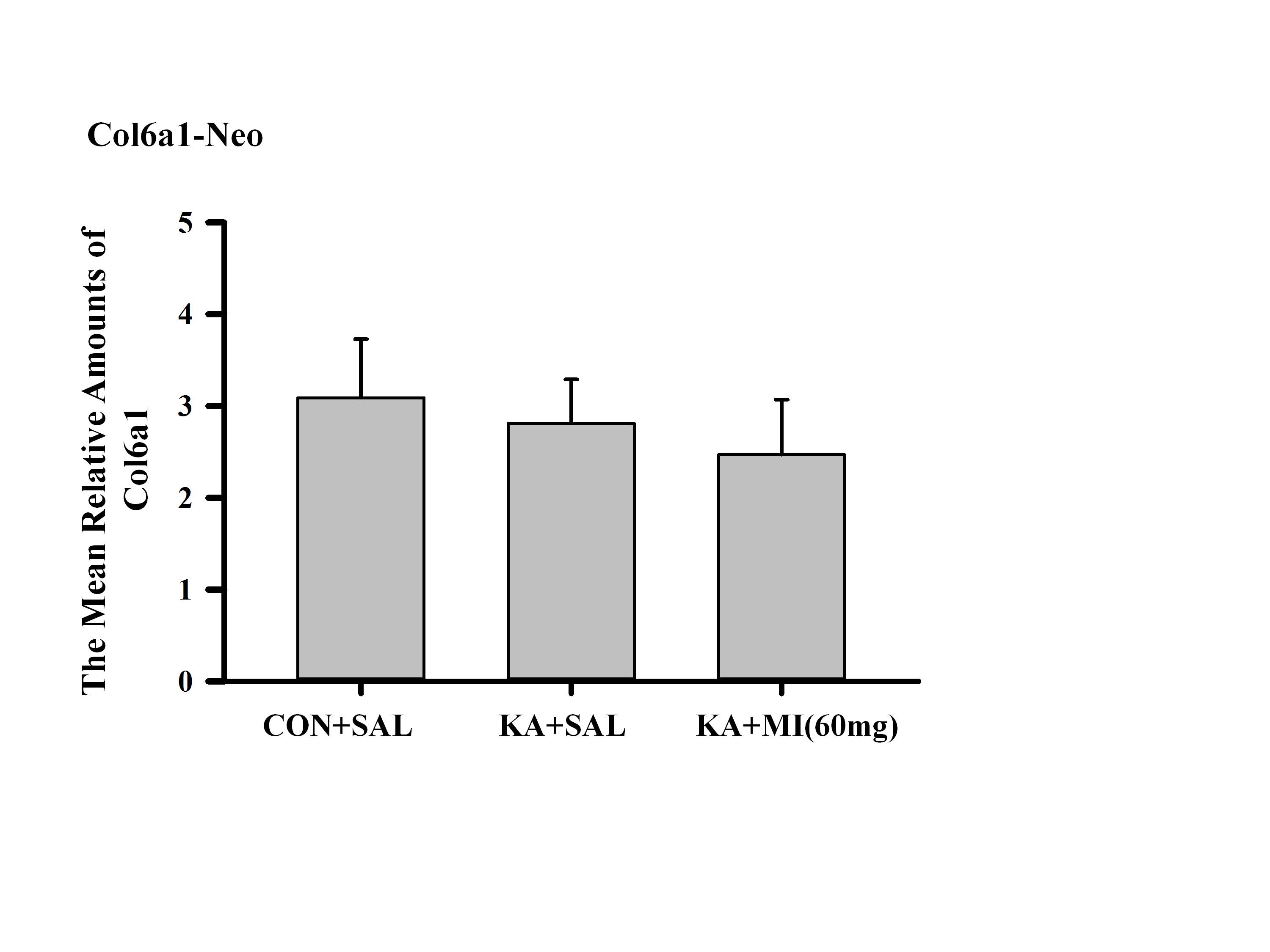

Supplement: Supplementary file 1 [file ijms-26-11102-s001.zip › Supplementary Figure S7.jpg]

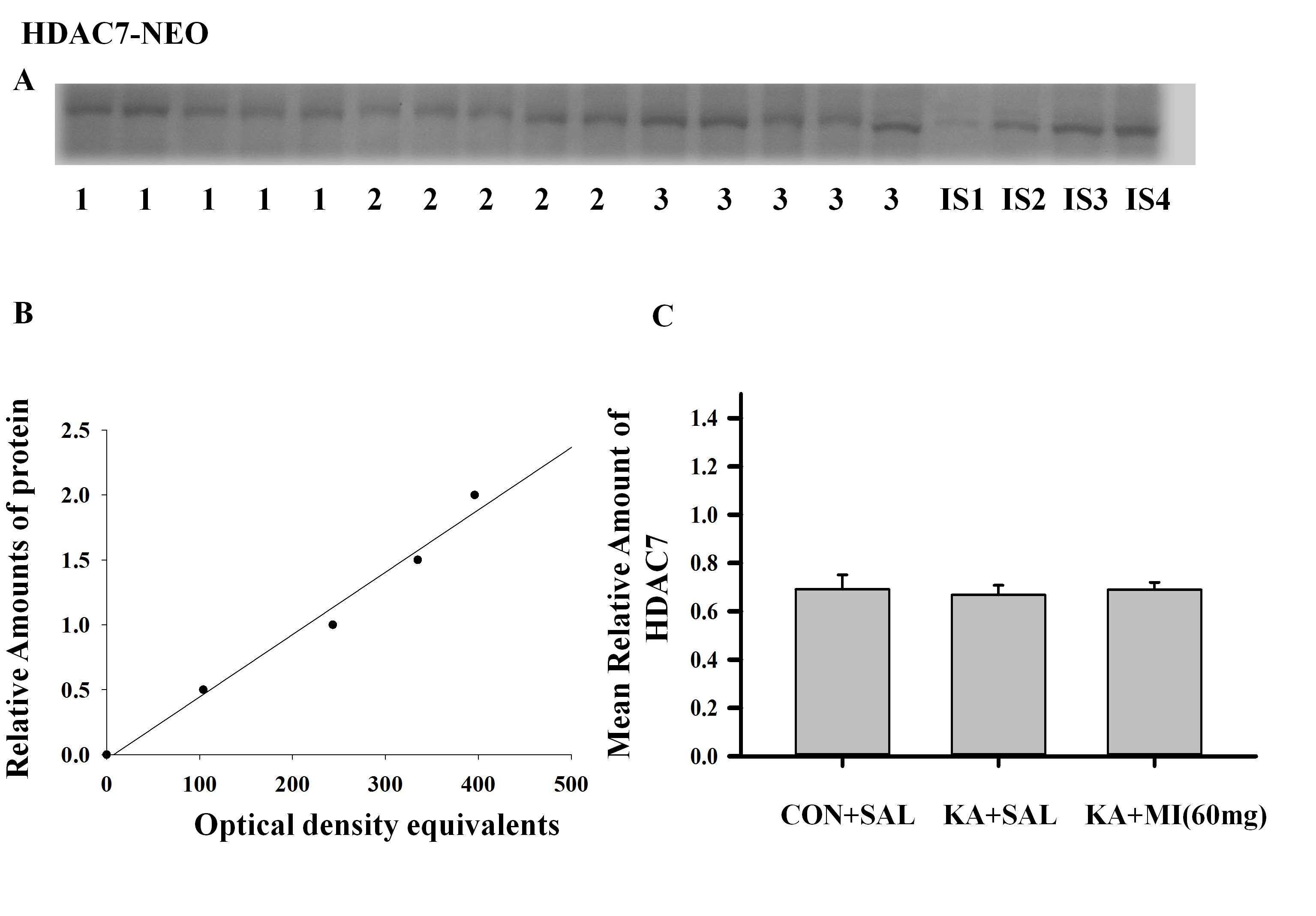

Supplement: Supplementary file 1 [file ijms-26-11102-s001.zip › Supplementary Figure S8.jpg]

## Ponceau Staining Images

HDAC7 Hippocampus

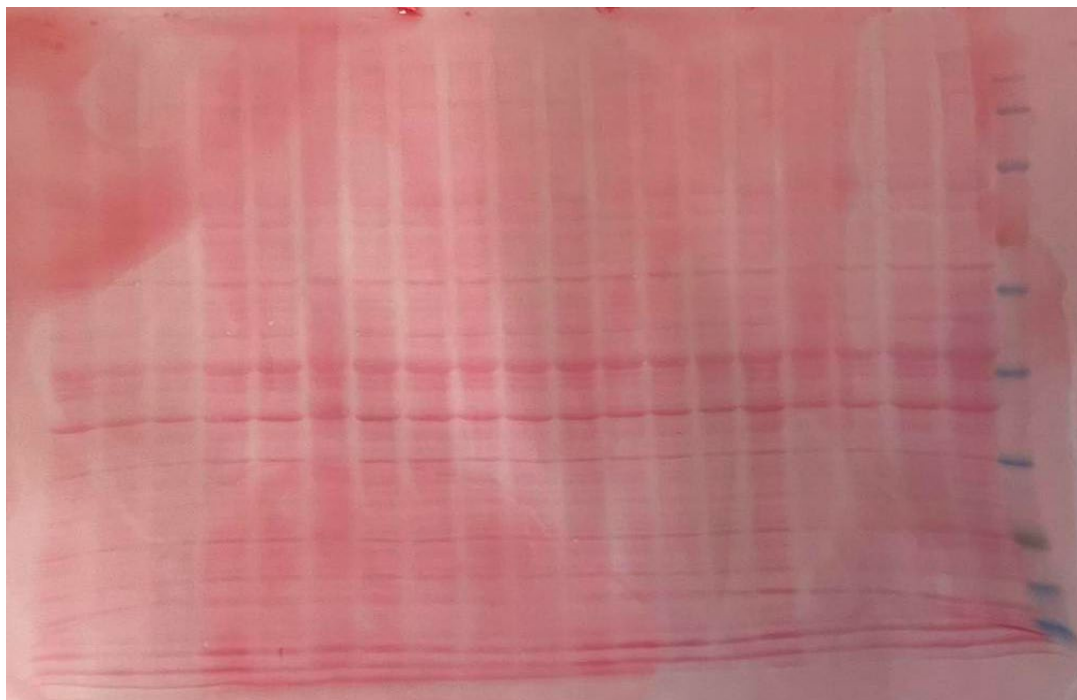

HDAC7 Neocortex

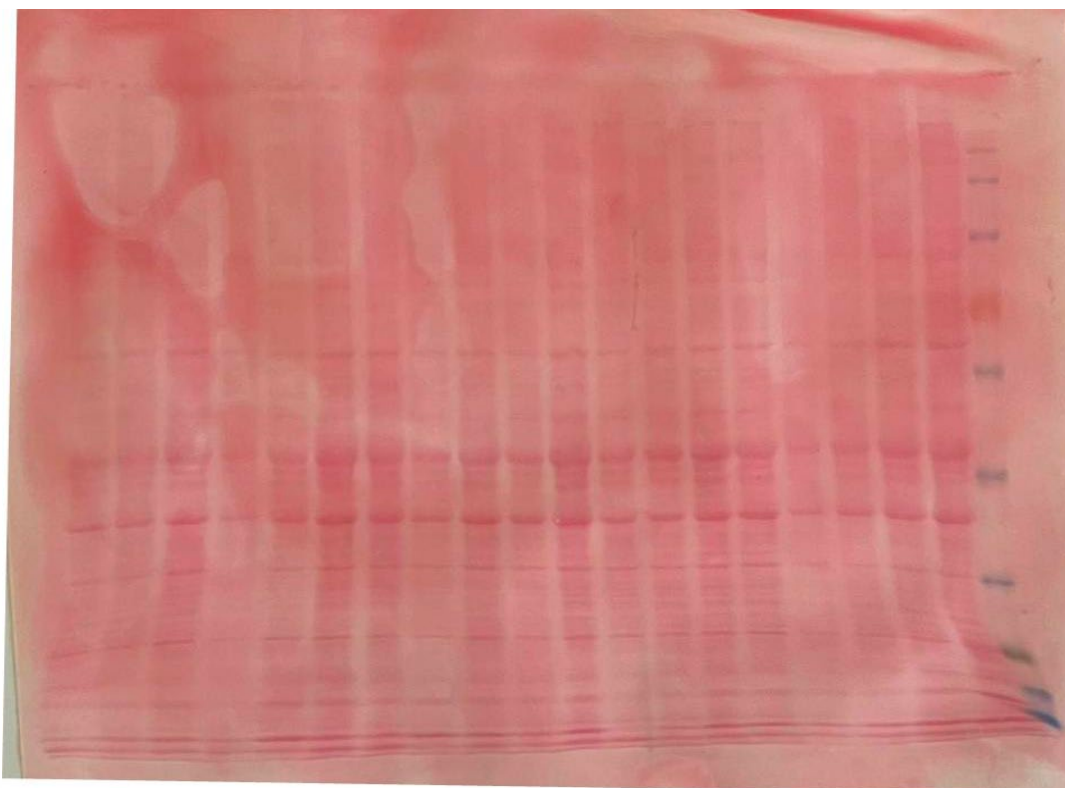

## HDAC7- Hippocampus

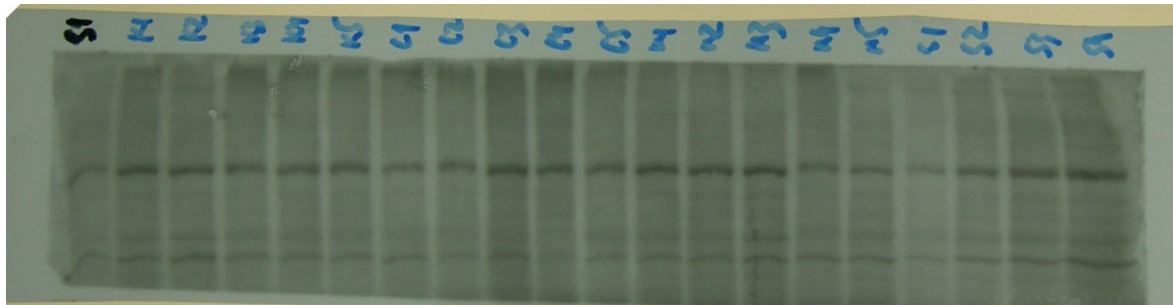

## HDAC7- Neocortex

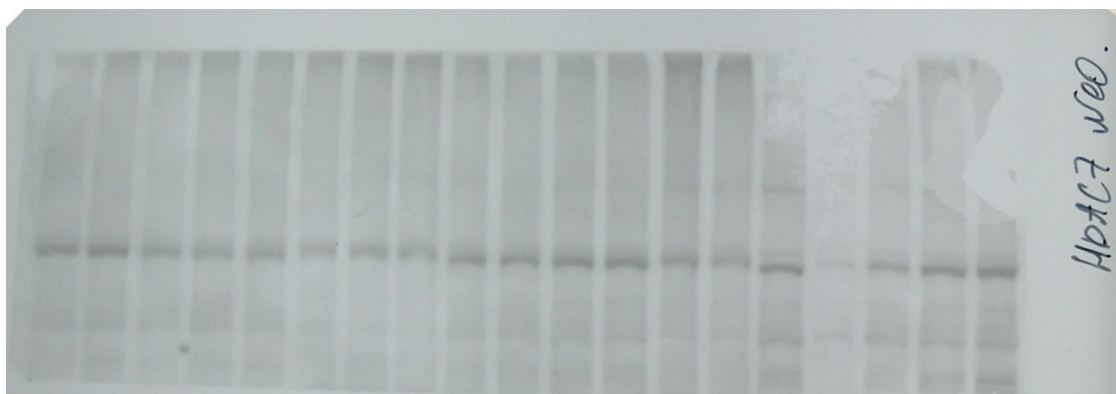

Supplement: Supplementary file 1 [file ijms-26-11102-s001.zip › Supplementary Figure S9 Ponceau staining images and uncropped blotes.pdf]
